# The NITRATE-OCT study-inorganic nitrate reduces in-stent restenosis in patients with stable coronary artery disease: a double-blind, randomised controlled trial

**DOI:** 10.1016/j.eclinm.2024.102885

**Published:** 2024-10-18

**Authors:** Krishnaraj S. Rathod, Anthony Mathur, Asad Shabbir, Rayomand S. Khambata, Clement Lau, Anne-Marie Beirne, Ismita Chhetri, Mutsumi Ono, Djouhar R. Belgaid, Gianmichele Massimo, Anantharaman Ramasamy, Vincenzo Tufaro, Ajay K. Jain, Neil Poulter, Emanuela Falaschetti, Daniel A. Jones, Hector M. Garcia-Garcia, Christos Bourantas, Anna Learoyd, Helen R. Warren, Amrita Ahluwalia

**Affiliations:** a Barts and the London Faculty of Medicine and Dentistry, Queen Mary University of London, London, United Kingdom; b Barts Heart Centre, St. Bartholomew's Hospital, London, United Kingdom; c Imperial College Trials Unit, London, United Kingdom; d MedStar Washington Hospital Center, Washington DC, USA

**Keywords:** Coronary, Dietary, In-stent, Nitric oxide, PCI, Restenosis

## Abstract

**Background:**

Coronary angioplasty and stent insertion is a first line treatment for patients with coronary artery disease, however it is complicated in the long-term by in-stent restenosis (ISR) in a proportion of patients with an associated morbidity. Despite this, currently there are no effective treatments available for the prevention of ISR. Repeat percutaneous revascularisation carries increased risks of major adverse cardiovascular events and a higher incidence of stent failure. In this study we report the efficacy of dietary inorganic nitrate in the prevention of ISR in a prospective, double-blind, randomised controlled trial.

**Methods:**

NITRATE-OCT is a double-blind, randomised, single-centre, placebo-controlled phase II trial. 300 patients who were planned to undergo percutaneous coronary intervention (PCI) and drug eluting stent (DES) implantation for stable angina were randomised on a 1:1 basis to receive a daily dose of either dietary inorganic nitrate or placebo for 6 months. Block randomisation was used and patients stratified according to diabetes status. The patients then underwent quantitative coronary angiography (QCA) at baseline and at 6 months and optical coherence tomography at 6 months to quantify ISR. The primary endpoint was the QCA quantified decrease of in-stent/in-segment diameter from the baseline measure at 6 months i.e., in-stent and in-segment late-lumen loss (LLL). The study is registered with ClinicalTrials.gov, number NCT02529189.

**Findings:**

From November 1st 2015 and March 31st 2020, NITRATE-OCT enrolled 300 patients with angina, with 150 each randomised to receive 70 mL of nitrate-containing beetroot juice or placebo (nitrate-deplete) juice for 6 months. Procedural characteristics were similar between the groups. The primary endpoint was available in 208 patients: 107 and 101 in the nitrate and placebo groups, respectively. There was a statistically significant effect of inorganic nitrate on both primary endpoints: in-stent LLL decreased by 0.16 mm (95% CI:0.06–0.25; P = 0.001) with mean = 0.09 ± 0.38 mm in the inorganic nitrate group versus 0.24 ± 0.33 mm in the placebo group; (P = 0.0052); and in-segment LLL decreased by 0.24 mm (95% CI:0.12–0.36; P < 0.001) with mean = 0.02 ± 0.52 mm in the inorganic nitrate group and 0.26 ± 0.37 mm in the placebo group (P = 0.0002). Inorganic nitrate treatment was associated with a rise in the plasma nitrate concentration of ∼6.1-fold and plasma nitrite (NO_2_^-^) of ∼2.0-fold at 6 months. These rises were associated with sustained decreases in systolic blood pressure (SBP) at 6 months compared to baseline with a change SBP of −12.06 ± 15.88 mmHg compared to the placebo group of 2.52 ± 14.60 mmHg (P < 0.0001).

**Interpretation:**

In patients who underwent PCI for stable coronary artery disease, a once-a-day oral inorganic nitrate treatment was associated with a significant decrease in both in-stent and in-segment LLL.

**Funding:**

This trial and KSR was funded by the 10.13039/501100000272National Institute for Health and Care Research (NIHR) (DRF-2014-07-008) and NIHR ACL, HW and this study were supported by The NIHR Barts Biomedical Research Centre, IC was funded by The North and East London Clinical Research Network, CL, GM were funded by The Barts Charity Cardiovascular Programme MRG00913 and MO was funded by The British Heart Foundation Project Grant PG/19/4/33995.

**Research in context**

**Evidence before this study**

In the drug eluting stent era effective treatments preventing in-stent restenosis (ISR), have not been forthcoming. Endothelial dysfunction, and thus deficiency of nitric oxide (NO), resulting in increased inflammation, smooth muscle hyperplasia and thrombosis is thought to play a key role in ISR. Despite this, studies indicate that organic nitrates, that deliver nitric oxide, are not efficacious. In part, this failure has been attributed to the development of tachyphylaxis to the organic nitrates. We tested whether these issues with organic nitrate therapy can be overcome using dietary delivery of inorganic nitrate that is converted to NO by the entero-salivary circuit.

**Added value of this study**

This study investigated the efficacy of dietary inorganic nitrate as a means to deliver nitric oxide, in reducing the rates of ISR in patients undergoing percutaneous coronary intervention (PCI) for chronic coronary syndrome. NITRATE-OCT demonstrated that once a day dietary inorganic nitrate reduces late-lumen loss significantly at 6-months, associated with reductions in blood pressure and increases in blood, urine, and saliva nitrate and nitrite levels.

**Implications of all the available evidence**

This study demonstrates that a simple once-a-day easy to administer inorganic nitrate dose reduces ISR which may translate to significant clinical benefits in larger studies.

## Introduction

Coronary artery disease (CAD) remains one of the most common causes of mortality in the UK^1^ and globally in both men and women.^2^ Percutaneous coronary intervention (PCI) in the setting of acute coronary syndrome (ACS) offers prognostic benefit to patients through the reduction of infarct size and preservation of ventricular function,^3^, ^4^, ^5^ and improves symptoms and quality of life in patients suffering from angina with chronic coronary syndrome.^6^^,^^7^ However, despite refinement in PCI techniques in conjunction with drug-eluting stents, recent evidence from the largest study to date (>5 million people in the USA) assessing outcomes post PCI + stent indicates that occurrence of in-stent restenosis (ISR) is substantial (∼5–10% over 8 years^8^ and even higher rates up to 20% at one year in high risk patients such as those with diabetes mellitus, chronic kidney disease or those with complex lesions). Moreover, these individuals experience greater co-morbidities and approximately 1 in 4 presents with an acute myocardial infarction (MI). In a separate meta-analysis including over 25,000 patients these events were shown to occur within the first five years post intervention.^9^ Since it is estimated that following PCI and stent insertion patients go on to live for 15 years and more,^10^ there is clearly a substantial unmet need. ISR is the consequence of progressive development of neo-intimal tissue, plaque and/or calcification within the region of the implanted stent; a process driven, in part, by unopposed inflammation, smooth muscle cell growth and platelet activation,^11^, ^12^, ^13^ and results in luminal re-stenosis and myocardial ischaemia. It is this process that is thought to underlie most of the long-term adverse event accrual rate.^9^

Whilst systemically delivered pharmacological treatments have been trialled previously with the aim of reducing the progression of ISR^14^, ^15^, ^16^, ^17^ there has been little success. To date only anti-platelet agents have shown clinical efficacy in preventing stent failure through prevention of thrombosis.^18^^,^^19^ Therefore, strategies to reduce the burden of ISR and thus potentially long-term events are needed.

Nitric oxide (NO), generated via the canonical l-arginine/NO synthesis pathway, is essential in maintaining vascular homeostasis.^20^^,^^21^ In the setting of CAD, endothelial dysfunction results in the depletion of NO,^22^ and thus loss of its cardioprotective functions as a potent vasodilator,^23^ anti-inflammatory,^24^^,^^25^ anti-platelet,^23^^,^^26^^,^^27^ and anti-proliferative mediator.^23^^,^^28^ Thus, restoration of NO in the setting of CAD might offer therapeutic potential in reducing adverse event risk. In addition to the l-arginine/NO pathway, endogenous NO can be augmented through activation of the ***non-canonical*** pathway for NO synthesis, from the *in vivo* reduction of inorganic nitrate (NO_3_^-^) to nitrite (NO_2_^-^) to NO in the body.^29^ In pre-clinical models of cardiovascular disease, inorganic nitrate or nitrite delivery reduces ischaemia-reperfusion injury,^30^^,^^31^ is anti-inflammatory in the setting of atherosclerosis^32^ and inhibits vascular smooth muscle cell hyperplasia following vascular damage.^33^ Utilisation of the non-canonical pathway for the generation of NO is appealing, since this pathway is not dependent upon enzymes that are dysfunctional in CAD and sustained activation of this pathway does not exhibit tachyphylaxis; a major limitation of the organic nitrates.^34^ We have previously demonstrated that circulating nitrite can be supplemented in humans through the consumption of inorganic nitrate-rich vegetables, including beetroots. Moreover, inorganic nitrate supplementation, results in NO-mediated improvements in blood pressure (BP),^35^, ^36^, ^37^ platelet^38^ and endothelial function in patients with cardiovascular disease.^39^ Since inflammatory and thrombotic pathways have been identified as key mechanisms in the development of ISR in patients we postulated that supplementing NO via the ***non-canonical*** pathway may provide a simple method to improve long term events in patients undergoing PCI.

Thus, this trial was designed to test whether dietary inorganic nitrate, on top of optimal medical therapy, leads to a reduction in intimal hyperplasia, and thereby prevents ISR as measured by angiographic-determined late-lumen loss (LLL) in patients with chronic stable coronary disease treated with PCI and drug eluting stent insertion for stable angina.

## Methods

### Study design

NITRATE-OCT was a single-centre, randomised double-blind trial performed in the UK at Barts Health National Health Service (NHS) Trust, within the Barts Heart Centre, St Bartholomew's Hospital, London. The first patient was recruited on November 1st 2015 with the final patient follow-up completed on March 31st 2022. A trial steering committee provided oversight of the study, and independent data monitoring was performed. A summary study protocol is available^40^ and the statistical analysis plan was concluded prior to analysis of data. Planning and oversight of the trial were provided by the UKCRC accredited Imperial Clinical Trials Unit and The Barts Cardiovascular Clinical Trials Unit.

### Ethics

The London City Road and Hampstead Research Ethics Committee (reference 15/LO/0555) approved the study and it is registered at ClinicalTrials.gov with Identifier: NCT02529189. Informed written consent was obtained from all patients prior to inclusion in the study. The trial was conducted according to the ethical framework of Barts Health NHS Trust and Queen Mary University of London Joint Research Management Office, in agreement with the Declaration of Helsinki.

### Patients

Patients were deemed suitable if they met the following inclusion criteria.

- ***stable*** angina diagnosed by a cardiologist and on optimal medical therapy (OMT),
- scheduled to undergo PCI and stent implantation,
- aged 18–85,
- able and willing to give written informed consent.

Exclusion criteria were: unstable ischaemic heart disease with an episode of chest pain at rest in <24 h before inclusion into the study, previous coronary artery bypass surgery if they were undergoing angioplasty within a non-native vessel, patients undergoing angioplasty with a bioabsorbable stent, diagnosis of or treatment for malignancy other than non-melanoma skin cancer, current life-threatening condition other than vascular disease that may prevent a participant completing the study, use of an investigational device or investigational drug within 30 days or five half-lives (whichever was longer) preceding the first dose of study intervention, patients considered unsuitable to participate by the research team (e.g., due to medical reasons, laboratory abnormalities or patient's unwillingness to comply with all study-related procedures), severe acute infection or significant trauma (burns, fractures), pregnancy tested by urine human chorionic gonadotropin measurement, history of alcohol or drug abuse within the past 6 months, a history of heart failure New York Heart Association (NYHA) class 3–4 or severe left ventricular dysfunction left ventricular ejection fraction <30% regardless of symptom status, systemic autoimmune disease such as rheumatoid arthritis, connective tissue disease or other conditions known to be associated with chronic inflammation such as inflammatory bowel disease, patients who have donated >500 mL blood within 56 days prior to study intervention administration, anaemia with haemoglobin <10 g/dL (6.21 mmol/L), or any other known blood disorder or significant illness that may affect platelet function, and coagulation, a history of chronic viral hepatitis or HIV, abnormal liver function due to acute or chronic liver conditions 3x upper limit of normal at screening, and renal impairment with creatinine clearance (estimated glomerular filtration rate) of 35 mL/min or less at screening.

### Protocol

**Fig. 1:**
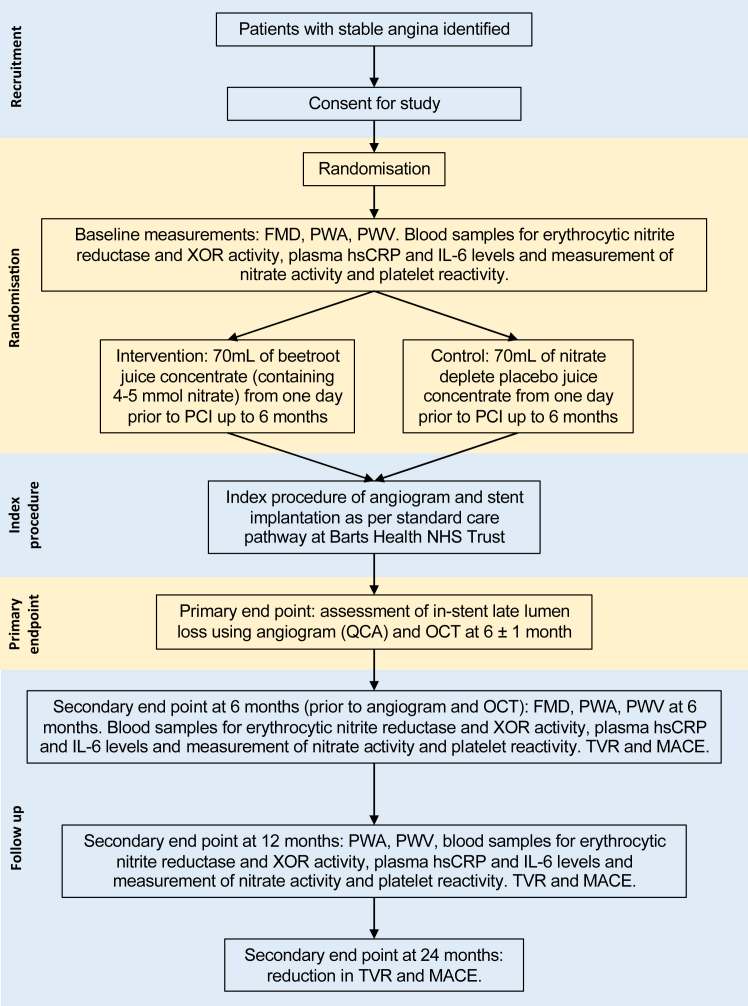
NITRATE-OCT study design. FMD, flow mediated dilatation; hsCRP, high-sensitivity C-reactive protein; IL-6, interleukin-6; MACE, major adverse cardiovascular event; OCT, optical coherence tomography; PCI, percutaneous coronary intervention; PWA, pulse wave analysis; PWV, pulse wave velocity; QCA, quantitative coronary angiography; TVR, target vessel revascularisation; XOR, xanthine oxidoreductase.

The study scheme is shown in Fig. 1. Following informed consent, biological sex was recorded as per the NHS record and baseline vital observations including pulse rate, BP, oxygen saturations, and temperature were recorded. Patient height and weight were measured to determine body-mass index. Peripheral venous blood samples were taken for haematological and biochemical clinical laboratory measurements including measurement of nitrite and nitrate. In addition, as per the protocol, several further mechanistic measurements were made including assessment of vascular flow-mediated dilatation (FMD), pulse wave analysis (PWA) and pulse wave velocity (PWV), in addition to a range of circulating cell analysis that are not presented here.

Following randomisation to either a daily 70 mL beetroot juice shot containing nitrate (James White Drinks, UK) from one day prior to PCI until 6 ± 1 months, or a nitrate-deplete placebo juice shot, patients underwent PCI. The dose chosen for this study was based upon numerous studies that have demonstrated that dietary inorganic nitrate causes dose-dependent rises in both nitrate and nitrite associated with dose-dependent beneficial effects in healthy volunteers and patients with various diseases including hypertension and hypercholesterolemia. Efficacious doses of inorganic nitrate sit close to the ADI as set by the European Food Standards Agency and the WHO.^29^^,^^41^ The placebo is identical in smell, taste and look and its preparation has been described previously and it has used extensively in clinical studies,^35^^,^^36^ with the full details available in the published protocol.^40^ For the primary endpoint coronary angiography was conducted at baseline and at 6 ± 1 months (using the same projections at both timepoints) for the primary end-points which were determined from independent blinded offline quantitative coronary angiography (QCA). A key mechanistic endpoint was optical coherence tomography (OCT) imaging of the treated vessel performed at a single timepoint of 6 ± 1 months after the index PCI procedure. Patients returned for an out-patient clinic visit at 12 months for peripheral venous blood samples for mechanistic biochemical outcome determination. Follow-up at 24 months was via the telephone.

### Randomisation and masking

In all patients, randomisation occurred prior to undertaking the index PCI. Patients were randomised on a 1:1 basis to receive a single daily dose of either dietary inorganic nitrate or placebo one day prior to PCI and daily thereafter for 6 months post-PCI. Block randomisation was used and patients stratified according to diabetic status. Diabetes was the only factor used for stratification and the randomisation list was created at the beginning of the study using the on-line randomisation tool www.random.org.

Treatment allocations were blinded to the patients, study team members, and clinical operators. An unblinding protocol was available, and treatment allocations were securely stored in a safe location in the William Harvey Heart Centre, Queen Mary University of London.

### Procedures

Blood, urine, and saliva were collected at baseline, 6-month, and 12-month timepoints. Venous blood samples were acquired to measure platelet activity, in addition to clinical haematology and biochemistry. Some blood samples were immediately centrifuged to generate platelet pellets and plasma collected for biochemical assessments (data to be presented in a separate mechanistic manuscript). Saliva and urine were collected in falcon tubes and stored at −80 °C for the analysis of nitrate and nitrite. A saliva sample was also acquired for the analysis of oral microbiota. Nitrate and nitrite concentrations were quantified using liquid phase ozone chemiluminescence.^42^ In addition, since nitrite reacts with oxy-haemoglobin, to generate met-haemoglobin and nitrate anion, we measured met-haemoglobin levels as an index for safety.

Diagnostic coronary angiography was performed at the time of the index PCI procedure either from radial or femoral access according to the operator's preference. The angiogram was performed after intracoronary administration of organic nitrate, and an effort was made to acquire projections that portrayed the treated lesion before and after PCI with minimal foreshortening or overlapping. PCI was then undertaken utilising techniques at the discretion of the operator. Baseline intracoronary imaging was permitted, as was the use of advanced plaque and calcium modification techniques. At 6 ± 1 months, repeat diagnostic coronary angiography was undertaken, with the same projections acquired from the baseline angiogram.

QCA data analysis was performed using a dedicated analysis software platform (Medis Suite version 3.2, Leiden, the Netherlands). An end-diastolic projection portraying the treated vessel after PCI was selected from the angiogram at baseline and the corresponding projection identified at follow-up and segmented by an experienced blinded analyst. The analyst identified the stented segment and its 5 mm proximal and distal edge and extracted the lumen borders using a dedicated edge detection algorithm -when required manual optimisation of the contouring was conducted. In each analysis the following metrics were computed: lesion length, reference vessel diameter computed using an interpolation technique, segment and the stented region (referred to as in-stent here onwards) minimum lumen area (MLA) and minimum lumen diameter (MLD), and the corresponding percentage diameter stenosis were calculated, with a mean calculated from the orthogonal projections. Lesion length, and proximal and distal reference vessel diameter were also acquired. In-segment LLL (defined as 5 mm proximal and distal to the stent) and in-stent LLL were defined as the difference between the post-procedure MLD and the MLD at 6 months. The analysis of the QCA was undertaken blind by two analysts, at the Barts Heart Centre Core lab (Rathod and Shabbir), and adjudicated by a third independent referee (CoreLab at MedStar Cardiovascular Research Network, Washington, DC, USA). This was performed to reduce both inter- and intra-observer variability.

OCT was performed at 6 ± 1 months after the index PCI procedure in all patients to establish a mechanistic explanation for any changes in LLL seen. The OCT catheter was advanced to the distal vessel—at least 10 mm distally to the distal end of the treated vessel and then pulled-back using automated device under contrast agent injection. The OCT images were obtained using the commercially available systems C7XR OCT acquiring 100 frames/s at a pullback speed of 20 mm/s and OPTIS acquiring 180 frames/s at a speed of 18–36 mm/s (St Jude Medical, Westford, MA, USA). Offline OCT image data was segmented using proprietary software (QCM-CMS, Leiden, the Netherlands). Analysis included the proximal and distal 5 mm stent edges and the stented segment and was performed at 1 mm interval. The OCT analysis was conducted by an independent CoreLab at MedStar Cardiovascular Research Network, Washington, DC, USA.

### Outcomes

The prespecified primary endpoint of the study was the reduction of in-stent LLL, assessed by QCA at 6 ± 1 months. Secondary endpoints included in-segment LLL, target vessel revascularisation (TVR) based on clinical judgement, restenosis rate (diameter >50%) and time-to-first event for major adverse cardiac events (MACE i.e., myocardial infarction, death and TVR) and nitrate and nitrite analysis of plasma, saliva and urine compared between the groups. In addition, a number of mechanistic endpoints assessing vascular function, systemic inflammation and plaque composition as assessed by OCT were included, and will be reported separately.

### Statistics

Sample size was determined for a total of 220 patients entering a two-treatment parallel-design study. The probability is 80 percent that the study will detect a treatment difference at a two-sided 5% significance level, if the true difference in LLL between the treatments is a conservative 0.22 mm based upon the variation (0.1–0.7 mm) as stated, at the time of sample size determination, in the large meta-analysis published by Mauri et al. This assumes that the standard deviation of the response variable is 0.550. This latter value is the mean of the standard deviations of 22 trials measuring LLL and showing an association of LLL (0.1–0.7 mm) with extent of angiographic determination of binary restenosis as described by Mauri et al.^43^ In addition, for an exploratory estimation of MACE in the elective angioplasty setting we based our sample size upon a study, in a similar cohort, testing the impact of ischaemic preconditioning in 215 patients with CCS who had undergone elective PCI for angina. In this study the intervention resulted in a reduction of MACE at 6 months to 3.6% versus 12.5% in the control arm.^44^ Using these data we estimated that 230 patients are required for 80% power using one-tailed analysis for a similar effect size. To account for dropouts for both outcomes, including non-compliance/withdrawals and loss due to change in cath lab operator decision making an overall 25% dropout rate was applied and a final target recruitment of 300 patients was estimated.

Statistical analysis was conducted both on an intention-to-treat (ITT) and a per protocol (PP) basis and performed on SPSS version 19 (SPSS Inc., Chicago, USA) GraphPad™ Prism v9 and Stata/MP v18.0.

Categorical data were summarised using absolute values (percentage). Continuous data were presented as mean ± SD or median (interquartile range if non-normally distributed). Continuous data were compared using Student's t-tests. Categorical data were compared using the Pearson chi-square test. Continuous data were compared between subgroups using Student's t-tests or Mann–Whitney tests if non-normally distributed.

Following independent CoreLab assessment of all angiograms, paired baseline and 6-month QCA data was available for 107 of the 139 patients who received a stent in the intervention group and 101 of the 135 in the placebo. The reasons for excluding angiograms are described below. Analysis of the association of treatment group with in-stent LLL and in-segment LLL was performed using linear regression, with covariate adjustment for diabetes status and MLD measured at baseline. Model assumptions were checked using residual and Q–Q plots. Secondary analyses adjusting for additional covariates, stratified by baseline organic nitrate use (as part of routine therapy) and examining the per-protocol population are described in the supplement.

For MACE the ITT data was analysed (150 in each group) and a second PP analysis was conducted in only those patients who had received a stent (i.e., 138 in the intervention arm and 134 in the placebo arm and due to operator decision on the day of the procedure). Kaplan–Meier analyses were performed for time-to-first event end points for major adverse cardiac events (i.e., myocardial infarction, death and TVR) and differences were tested using the log-rank test.

The NITRATE-OCT study is registered with ClinicalTrials.gov, number NCT02529189 and International Standard Randomised Controlled Trials Number (ISRCTN) 17373946. The protocol for the study was peer-reviewed and published.^40^

### Role of the funding source

The funder of the study (NIHR) had no role in study design, data collection, data analysis, data interpretation, or writing of the report.

## Results

### Clinical characteristics of patients

**Fig. 2:**
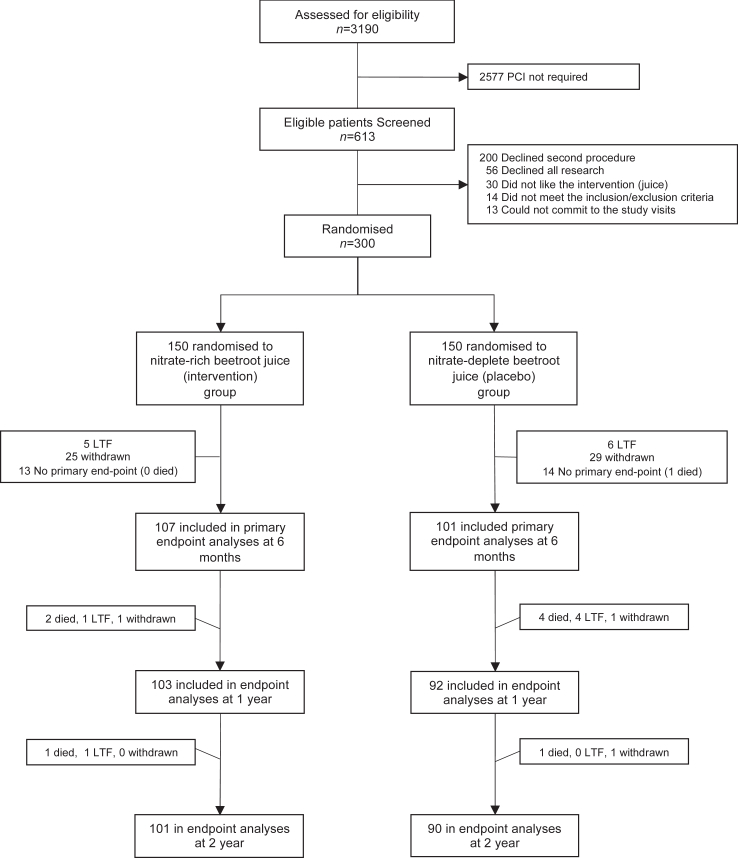
Trial consort diagram. LTF, lost to follow up.

Between November 1st 2015 and March 31st 2020, 3190 patients with angina attending the Barts Heart Centre were assessed for eligibility (Fig. 2). Of these 613 patients were listed by their cardiologist for angioplasty and stent insertion and were therefore screened for this study. Of the patients screened, 313 were excluded (200 declined participation due primarily to no desire to undergo a second procedure at 6 months, 56 declined to take part in research, 30 did not want to take the intervention, 14 did not meet the inclusion/exclusion criteria on further assessment and 13 could not commit to the study visits). Of these patients approximately 13% were female a proportion, whilst slightly lower than recent assessments,^45^ reflect an expected lower proportion based upon patients attending cardiac services in the UK.

In total 300 patients (Table 1) were randomised. There were no differences between the treatment groups in the numbers of patients on any type of medical therapy and 22% of the patients overall had diabetes (Supplementary Appendix, Table S1). At enrolment 242 (80.7%) of the patients were experiencing at least Canadian Cardiovascular Society (CCS) class II angina (with the remaining experiencing CCS Class I). The left anterior descending artery was most frequent target vessel in both groups; 40.1% in the intervention group, and 49.3% in placebo (Table 1).

**Table 1:** Baseline characteristics of the NITRATE-OCT Cohort randomised to treatment once a day for 6 months in a 1:1 ratio into the dietary nitrate intervention or the placebo groups.

	Inorganic Nitrate (n = 150)	Placebo (n = 150)
Age (years) (mean ± SD)	61.61 ± 8.92	61.42 ± 9.75
Sex (Male)	130 (86.7%)	131 (87.3%)
Ethnicity		
Caucasian	119 (79.3%)	118 (78.7%)
African-Caribbean	10 (6.7%)	10 (6.7%)
East Asian	3 (2.0%)	2 (1.3%)
South Asian	18 (12.0%)	20 (13.3%)
Indian	11 (7.3%)	9 (6.0%)
Bangladeshi	2 (1.3%)	1 (0.7%)
Pakistani	5 (3.3%)	10 (6.7%)
Diabetes mellitus	33 (22.0%)	33 (22.0%)
Type I	1 (0.7%)	1 (0.7%)
Type II	32 (21.3%)	32 (21.3%)
Body-mass index (kg/m^2^) (mean ± SD)	28.80 ± 4.44	28.96 ± 4.77
Missing	2	3
Hypertensionv	110 (73.3%)	111 (74.0%)
Hypercholesterolaemia	104 (69.3%)	112 (74.7%)
Previous MI	55 (36.7%)	56 (37.3%)
Previous PCI	48 (32.0%)	54 (36.0%)
Previous CABG	6 (4.0%)	8 (5.3%)
Current Smoker	22 (14.7%)	33 (22.0%)
Previous Smoker	75 (50.0%)	64 (42.7%)
PVD	11 (7.3%)	10 (6.7%)
CVA/TIA	7 (4.7%)	7 (4.7%)
NYHA		
Class I	6 (4.1%)	10 (6.8%)
Class II	8 (5.5%)	6 (4.1%)
Class III	0	2 (1.4%)
Missing	5	3
CCS		
CCS I	28 (18.8%)	27 (18.1%)
CCS II	54 (36.2%)	52 (34.2%)
CCS III	65 (43.6%)	66 (45.0%)
CCS IV	1 (0.7%)	4 (2.7%)
Missing	1	1
Asthma	16 (10.7%)	14 (9.3%)
COPD	10 (6.7%)	8 (5.3%)
Previous History of CAD	75 (50.0%)	80 (53.3%)
Heart rate (BPM) (mean ± SD)	69.71 ± 14.03	66.64 ± 12.08
Missing	7	5
Systolic BP (mmHg) (mean ± SD)	137.20 ± 16.85	136.77 ± 16.86
Missing	9	8
Diastolic BP (mmHg) (mean ± SD)	78.94 ± 9.89	77.46 ± 10.13
Missing	9	8
Culprit Vessel		
Left main stem	0	0
Left anterior descending	55 (40.1%)	66 (49.3%)
First diagonal	4 (2.9%)	1 (0.7%)
Intermediate Artery	2 (1.5%)	3 (2.2%)
Circumflex	26 (19.0%)	19 (14.2%)
Obtuse Marginal	4 (2.9%)	4 (3.0%)
Right coronary	46 (33.6%)	41 (30.6%)
Missing	13	16

BP, blood pressure; BPM, beats per minute; CABG, coronary artery bypass graft; CAD, coronary artery disease; CCS, Canadian Cardiology Society angina classification; COPD, chronic obstructive airways disease; CVA, cerebrovascular accident; MI, myocardial infarction; PCI, percutaneous coronary intervention; PVD, peripheral vascular disease; SD, standard deviation; TIA, transient ischaemic attack. Sex status relates to biological sex at birth as per NHS records.

A total of 54 patients who consented and were included and underwent all clinical assessments at baseline withdrew from the study prior to the 6-month angiogram and thus the primary outcome was not available for these consented patients. Of these 27 patients stated that the juice caused gastrointestinal upset, and the remaining 27 patients stated they did not wish to undergo a second angiogram procedure. A further 11 patients were lost to follow up and 27 had no primary end-point due to not undergoing implantation with a stent at the time of the index procedure.

### Procedural characteristics

Procedural characteristics are shown in Table 2. Of the 300 ITT patients consented and randomised, only 138 in the inorganic nitrate group and 134 patients in the placebo group received the ‘planned’ stent implantation due to operator decisions made at the time of the procedure; an event accounted for in our sample size calculations. In those patients receiving a stent, 1 patient received a Bare Metal Stent (BMS) stent based upon operator choice (in the intervention arm and included in the ITT analysis). Of those who had a DES stent implanted (all latest generation), Xience stents (everolimus eluting stent) were used predominantly with 78.3% and 83.6% in the intervention and placebo groups respectively (see Table 2 for details of all other stents used). Radial access was used in most cases, with 82.7% in the intervention group, and 80.7% in the placebo group. The number of stents used in the intervention group was 1.39 ± 0.71, and 1.30 ± 0.52 in placebo. Stent length and diameter (of each stent) did not differ between the groups, with a mean length of the first stent implanted of 27.20 ± 9.83 mm in the intervention group, and 27.41 ± 9.96 mm in placebo, with a diameter of 3.17 ± 0.43 in intervention and 3.1 ± 0.45 mm in placebo. Procedural success (defined as TIMI 3 flow and an acceptable result on the final angiogram) was 100% in both groups. Choice of target lesion was at the discretion of the operator in this group of patients with stable symptoms.

**Table 2:** Procedural characteristics of patients recruited into the NITRATE-OCT trial.

	Inorganic Nitrate (n = 150)	Placebo (n = 150)
Anti-platelet therapy	150 (100%)	150 (100%)
Aspirin	150 (100%)	150 (100%)
Clopidogrel	123 (82.0%)	116 (77.3%)
Ticagrelor	27 (18%)	34 (22.7%)
Radial access	124 (82.7%)	121 (80.7%)
Medical Therapy	4 (2.7%)	14 (9.3%)
CABG	7 (4.7%)	1 (0.7%)
DEB Use	1 (0.7%)	1 (0.7%)
Stent implantation	138 (92.0%)	134 (89.3%)
DES use	137 (91.3%)	134 (89.3%)
Number of stents used (mean ± SD)	1.39 ± 0.71	1.30 ± 0.52
Stent Type		
Bare Metal Stent	1 (0.7%)	0
Xience	108 (78.3%)	112 (83.6%)
Resolute Integrity	5 (3.6%)	3 (2.2%)
Promus Premier	14 (10.1%)	9 (6.7%)
Biofreedom	2 (1.4%)	2 (1.5%)
Synergy	5 (3.6%)	7 (5.2%)
Biomatrix	1 (0.7%)	0
Unlisted	2 (1.4%)	1 (0.7%)
Stent length (mm, mean ± SD)		
First Stent	27.20 ± 9.83	27.41 ± 9.96
Second Stent	21.51 ± 8.65	23.12 ± 11.35
Third Stent	16.45 ± 7.54	13.25 ± 3.20
Fourth Stent	26.50 ± 16.26	
Total length of stent (mm, mean ± SD)	34.98 ± 17.75	33.51 ± 16.71
Proportion of stents		
First Stent	138 (92.0%)	134 (89.3%)
Second Stent	39 (26.0%)	33 (22.0%)
Third Stent	11 (7.3%)	4 (2.7%)
Fourth Stent	2 (1.3%)	0
Stent diameter (mm, mean ± SD)		
Overall	3.17 ± 0.43	3.12 ± 0.45
First Stent	3.10 ± 0.46	3.14 ± 0.46
Second Stent	3.26 ± 0.48	3.17 ± 0.49
Third Stent	3.27 ± 0.47	3.75 ± 0.28
Fourth Stent	3.00 ± 0.71	
Procedural Success in those receiving PCI	139/139 (100%)	135/135 (100%)

Data shown as mean ± SD, or percentage. DES, drug eluting stent; SD, standard deviation. Stent characteristics given apply to the index vessel only.

### Influence of inorganic nitrate on in-stent restenosis: primary outcome

Overall, angiograms were available for analysis in 107 of the 138 patients in the intervention arm and 101 of 134 in the placebo arm at both the baseline and 6-month timepoints. Quality control review by the Independent CoreLab led to exclusion of 8 angiograms in the intervention arm and 12 in the placebo arm prior to unblinding. The reasons for exclusion were: suboptimal opacification of the studied vessel/presence of the diaphragm (n = 11), multiple vessels overlapping (n = 6), more than 25° difference in the projections at 6 months compared to baseline (n = 1), presence of a guide wire (n = 1) and poor visibility of the stent (n = 1). This meant that there were angiograms of 99 (intervention arm) and 89 (placebo arm) patients included in the primary analysis. Baseline stent MLD (Table 3), reference vessel dimensions and stent length (Table 4) were not significantly different between groups as assessed by QCA. There were no differences in the absolute number of days post-PCI between the 1st and the 2nd angiogram (Intervention 186 (IQR: 175–215) versus Placebo 194: (181–213)) (P = 0.613).

**Table 3:** Intention to treat (ITT) analysis of the primary end-point using quantitative coronary angiography (QCA) of patients recruited into the NITRATE-OCT trial.

	Observed Mean ± SD		Comparison between treatments	
	Inorganic Nitrate (*n* = 107)	Placebo (*n* = 101)	Estimated difference (95% CI)	P value
			Unadjusted differences (t-tests)	
Baseline in-stent MLD (mm)	2.69 ± 0.50	2.65 ± 0.44	0.03 (−0.09, 0.16)	0.593
Diabetics	2.69 ± 0.48 (*n* = 25)	2.62 ± 0.49 (*n* = 18)		
Non-diabetics	2.69 ± 0.51 (*n* = 82)	2.66 ± 0.43 (*n* = 83)		
Baseline in-segment MLD (mm)	2.40 ± 0.57	2.39 ± 0.47	0.01 (−0.13, 0.15)	0.891
Diabetics	2.35 ± 0.58 (*n* = 25)	2.34 ± 0.46 (*n* = 18)		
Non diabetics	2.41 ± 0.57 (*n* = 82)	2.40 ± 0.48 (*n* = 83)		
Follow up in-stent MLD (mm)	2.59 ± 0.55	2.42 ± 0.45	**0.18 (0.04, 0.31)**	**0.013**
Diabetics	2.55 ± 0.45 (*n* = 25)	2.23 ± 0.49 (*n* = 18)		
Non-diabetics	2.60 ± 0.58 (*n* = 82)	2.46 ± 0.44 (*n* = 83)		
Follow up in-segment MLD (mm)	2.37 ± 0.63	2.13 ± 0.51	**0.25 (0.09, 0.41)**	**0.002**
Diabetics	2.37 ± 0.52 (*n* = 25)	2.0 ± 0.57 (*n* = 18)		
Non diabetics	2.38 ± 0.66 (*n* = 82)	2.1 ± 0.50 (*n* = 83)		
			Adjusted differences (linear regression models)	
In-stent late-lumen loss (mm)	0.09 ± 0.38	0.24 ± 0.33	**−0.16 (−0.25, −0.06)**	**0.001**
Diabetics	0.14 ± 0.30 (*n* = 25)	0.39 ± 0.29 (*n* = 18)		
Non-diabetics	0.08 ± 0.40 (*n* = 82)	0.20 ± 0.33 (*n* = 83)		
In-segment late-lumen loss (mm)	0.02 ± 0.52	0.26 ± 0.37	**−0.24 (−0.36, −0.12)**	**<0.001**
Diabetics	−0.02 ± 0.38 (*n* = 25)	0.31 ± 0.34 (*n* = 18)		
Non-diabetics	0.04 ± 0.55 (*n* = 82)	0.25 ± 0.38 (*n* = 83)		

Data shown as mean ± SD. P value calculated using Student's unpaired T test for MLD and linear regression models with covariate adjustment for baseline MLD and diabetes for late-lumen loss. The estimated difference between treatment groups relates to the difference in Inorganic Nitrate Treatment Group versus Placebo Group (reference group). Bold highlights statistical significance. CI, confidence interval. MLD, minimum lumen diameter.

**Table 4:** Intention to treat (ITT) analysis of other parameters from quantitative coronary angiography (QCA) of patients recruited into the NITRATE-OCT trial.

	Inorganic Nitrate (*n* = 107)	Placebo (*n* = 101)	P value
Baseline stent diameter (mm)	3.20 ± 0.47	3.14 ± 0.43	0.235
Baseline stent length (mm)	24.9 (17.9–35.8)	23.2 (18.3–33.6)	0.729
Baseline proximal reference stent diameter (mm)	3.39 ± 0.64	3.52 ± 1.52	0.768
Baseline distal reference stent diameter (mm)	2.66 ± 0.53	2.67 ± 0.50	0.844
Baseline in-stent diameter stenosis (%)	12.3 (7.8–16.3)	12.5 (8.1–17.4)	0.494
Baseline proximal maximum in-segment diameter (mm)	3.79 ± 0.77	3.86 ± 1.09	0.845
Baseline distal maximum in-segment diameter (mm)	2.94 ± 0.75	3.07 ± 0.71	0.210
Baseline in-segment diameter stenosis (%)	18.3 (12.0–25.0)	18.9 (12.2–24.5)	0.690
Follow up diameter stenosis in-stent (%)	14.5 (10.0–19.3)	14.9 (9.6–21.0)	0.817
Follow up diameter stenosis in-segment (%)	17.7 (11.3–25.0)	19.1 (12.0–26.0)	0.224

Data shown as mean ± SD or median (interquartile range) if non-normally distributed. P value calculated using Student's unpaired T test or Mann Whitney tests if non-normally distributed. MLD, minimum lumen diameter.

**Fig. 3:**
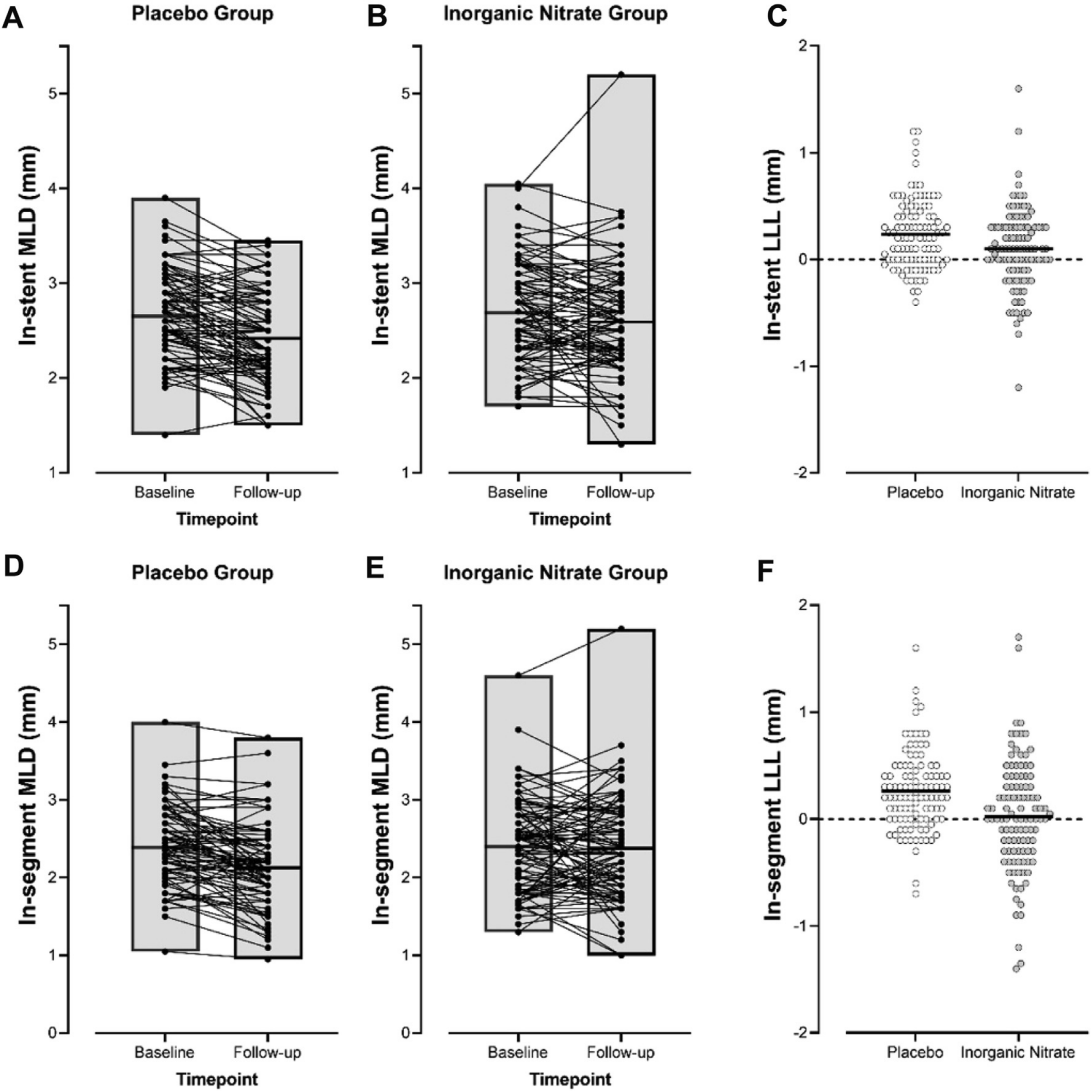
In-stent (A–C) and in-segment (D–F) minimum lumen diameter (MLD) and resultant late-lumen loss (LLL) determined using quantitative coronary angiography. Panels (A) and (B) show the in-stent MLD at both timepoints for patients on placebo and inorganic nitrate respectively. Panel (C) shows the resultant significant difference in in-stent LLL at 6 months follow-up in both treatment groups (P = 0.001). Panels (D) and (E) show the comparable data for in-segment MLD with panel (F) showing the resultant significant difference in in-segment LLL between treatment groups (P < 0.001). Lines on each panel indicate the mean at that timepoint/in that treatment group.

For the primary endpoint in-stent LLL was 0.09 ± 0.38 mm in the patients treated with the dietary inorganic nitrate intervention compared with 0.24 ± 0.33 mm in those receiving placebo (Fig. 3). After adjustment for diabetes and baseline stent MLD, this corresponds to a statistically significant effect, with inorganic nitrate treatment decreasing in-stent LLL by 0.16 mm (95% CI, 0.06–0.25; P = 0.001; Table 3). In-segment LLL was observed to be 0.02 ± 0.52 mm in the dietary inorganic nitrate intervention arm, and 0.26 ± 0.37 mm in patients treated with placebo (P = 0.0002). After adjustment for diabetes and baseline segment MLD, this corresponds to a statistically significant effect, with nitrate treatment decreasing in-segment LLL by 0.24 mm (95% CI, 0.12–0.36; P < 0.001). These differences remained significant after further adjustment in the fully-adjusted model (Supplementary Appendix Table S4). Sensitivity analysis accounting for patients with missing data indicated that if mean stent LLL differed by 0.2–0.3 between observed and missing patients in either group (but not both) then in-stent LLL would not significantly differ between treatment groups and if mean segment LLL differed by 0.4–0.5 between observed and missing patients in either group (but not both) then in-segment LLL would not significantly differ between treatment groups (Supplementary Appendix Tables S2, S3 & Figure S1). For pictorial representation of the cumulative distribution of in-stent LLL see Supplementary Appendix Figure S2 for the placebo (A) and intervention (B) arms. Per protocol analyses of the primary endpoint, which included only those scans deemed of sufficient quality by the independent CoreLab, still confirmed a significant decrease in the in-stent LLL and in-segment LLL following treatment with inorganic nitrate compared to the placebo group (Supplementary Appendix Table S6).

For a priori subgroup analyses, stratifying by organic nitrate use (as part of routine therapy), in-segment LLL remained significantly different between the treatment groups. The in-stent LLL association remained significantly different between the groups within patients taking organic nitrate (N = 121), but no longer significant in those who were not taking these drugs (N = 87), perhaps due to the smaller stratified sample size (Supplementary Appendix Table S5, Figure S3).

#### OCT analysis: secondary outcome

Analysis of the OCT images acquired at 6 months identified a statistically significant greater maximum lumen area of 9.9 ± 0.35 mm^2^ in patients treated with the dietary inorganic nitrate compared to those receiving placebo of 9.0 ± 3.1 mm (P = 0.046). No significant differences were observed in the absolute minimum, mean stent or lumen diameter, or neointimal volume between either group at 6 months (Table 5).

**Table 5:** Optical coherence tomography (OCT) of the stent region at 6 months following daily dietary inorganic nitrate intervention or placebo in patients recruited into the NITRATE-OCT trial.

	Inorganic Nitrate (*n* = 103)	Placebo (*n* = 94)	P value
No. of analysable in-stent frames	26 (19–33)	24 (20–32)	0.429
No. of analysable lumen frames	29 (20–40)	27 (21–38)	0.646
Minimum lumen diameter (mm)	2.23 ± 0.5	2.17 ± 0.4	0.449
Maximum lumen diameter (mm)	3.89 ± 0.7	3.75 ± 0.7	0.135
Mean lumen diameter (mm)	2.98 ± 0.5	2.90 ± 0.5	0.257
Minimum lumen area (mm^2^)	5.15 ± 2.1	4.84 ± 2.0	0.294
**Maximum lumen area (mm**^**2**^**)**	**9.93 ± 3.5**	**8.98 ± 3.1**	**0.046**
Mean lumen area (mm^2^)	7.25 ± 2.4	6.86 ± 2.4	0.265
Lumen volume (mm^3^)	198 (131–281)	188 (118–262)	0.205
Minimum in-stent diameter (mm)	2.52 ± 0.5	2.48 ± 0.4	0.487
Maximum in-stent diameter (mm)	3.89 ± 0.6	3.80 ± 0.6	0.290
Mean in-stent diameter (mm)	3.16 ± 0.5	3.08 ± 0.5	0.284
Minimum in-stent area (mm^2^)	6.18 ± 2.1	5.93 ± 2.0	0.404
Maximum in-stent area (mm^2^)	10.13 ± 3.1	9.56 ± 3.0	0.186
Mean in-stent area (mm^2^)	8.05 ± 2.4	7.68 ± 2.4	0.280
Total No of Struts	257 (191–374)	257 (195–338)	0.646
% Malapposed struts	0.52 (0.00–2.16)	0.61 (0.00–2.14)	0.767
Median tissue Neointimal Area (mm^2^)	0.74 (0.55–0.93)	0.69 (0.51–0.97)	0.646
Mean ISA (Incomplete Stent Apposition) distance (mm)	0.005 (0.001–0.017)	0.003 (0.001–0.017)	0.488
Maximum ISA area (mm^2^)	0.01 (0.00–0.78)	0.01 (0.00–0.40)	0.490
Mean ISA area (mm^2^)	0.002 (0.000–0.028)	0.002 (0.000–0.030)	0.650
Total ISA area if malapposition is present (mm^2^)	0.006 (0.002–0.130)	0.006 (0.002–0.072)	0.722
% Neointimal hyperplasia	9.70 (7.14–13.91)	9.80 (9.09–13.14)	0.968
Neointimal volume (mm^3^)	27.4 ± 20.9	25.1 ± 18.1	0.407
Neointimal thickness (mm)	0.11 ± 0.05	0.11 ± 0.05	0.918
Maximal Neointimal obstruction (mm^2^)	1.92 ± 1.19	1.80 ± 1.08	0.455
Mean Neointimal obstruction (mm^2^)	0.81 ± 0.39	0.79 ± 0.41	0.717

Data shown as mean ± SD or median (interquartile range) if non-normally distributed. P value calculated by Student's unpaired T test or Mann Whitney tests if non-normally distributed. Calculated values: Neointimal Area = the stent inner surface area minus the total in-stent lumen area, % Neointimal hyperplasia = the neointimal area/stent area x100. Bold representing parameter showing statistical significance.

### 6 month, 1 year and 2 year follow-up: MACE

**Fig. 4:**
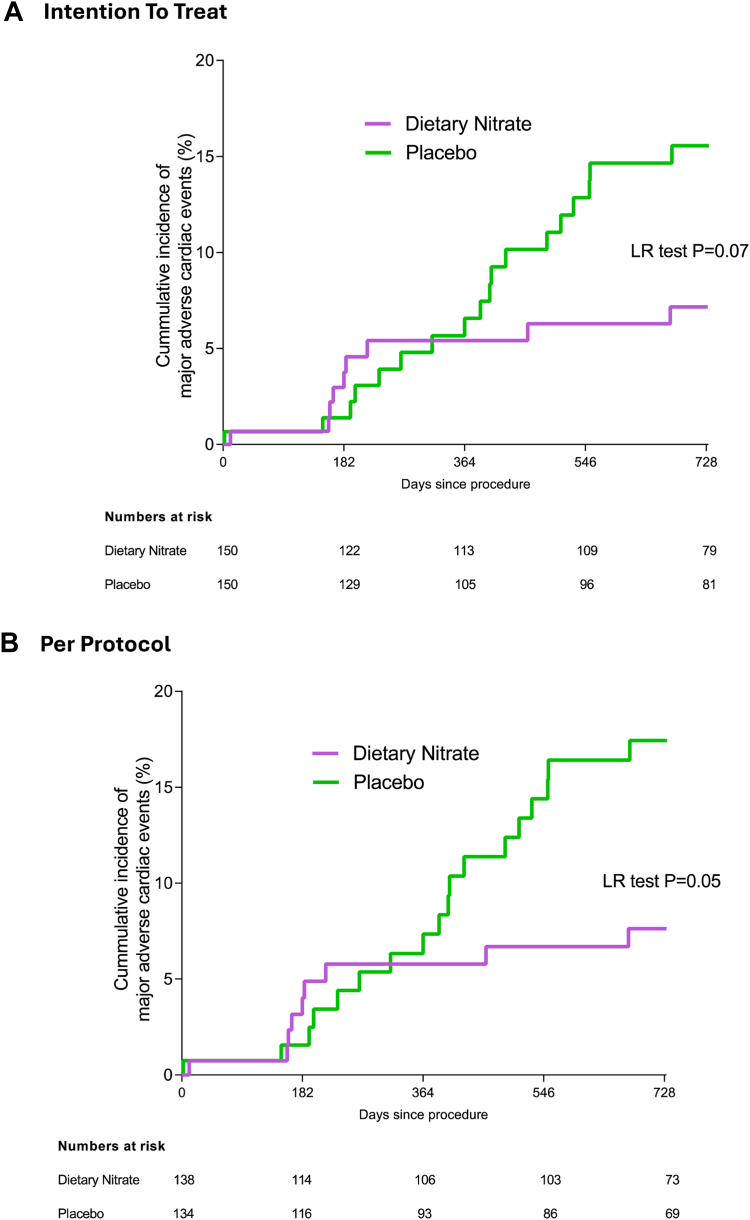
The cumulative incidence of MACE over 2 years in patients recruited into the NITRATE-OCT trial is demonstrated as time to first event between the two groups using (A) intention to treat analysis and (B) per protocol analysis including only those receiving a DES stent. Statistical significance is demonstrated using a Log–Rank test. MACE, major adverse cardiac events.

Whilst at 6 months MACE rates were no different between the groups, at 1 year and 2 years MACE rates in those receiving inorganic nitrate were significantly reduced in comparison to placebo, in those patients who received a stent (Supplementary Appendix Table S7, Fig. 4). Overall, at 2 years following PCI and stent insertion 27 patients experienced a MACE (7 deaths in total with 2 in the intervention group and 5 in the placebo group), 7 recurrent myocardial infarction (1 in the intervention group and 6 in the placebo group) and 13 unscheduled revascularisations (5 in the intervention group and 8 in the placebo group)). Overall, in the intention to treat analysis there was a trend towards a reduction in MACE events in the intervention group compared to the placebo group (P = 0.07 (Fig. 3a). However, in the per protocol analysis, excluding all patients who did not have a stent, a significant decrease in MACE in the inorganic nitrate intervention group compared to the placebo group was observed (P = 0.049) (Fig. 4b).

### Influence of inorganic nitrate on BP in patients with CAD

The inorganic nitrate intervention and placebo were both tolerated well. A summary of the adverse and serious adverse events is given in Table 6. In the inorganic nitrate treated group, SBP reduced by 12.06 ± 15.88 mmHg at 6 months compared to baseline. Meanwhile in the placebo group SBP increased by 2.52 ± 14.60 mmHg. Overall change in SBP differed significantly between groups by an estimated −14.58 mmHg (−18.66 to −10.49) (P < 0.0001) (Supplementary Appendix Figure S4A). A similar effect was evident in diastolic blood pressure (DBP) with an estimated reduction in change of DBP of 6.65 mmHg (4.20–9.10) in the inorganic nitrate group compared to placebo (P < 0.001) (Supplementary Appendix Figure S4B). In addition, a small (estimated at −2.80 bpm (−4.72 to −0.89)) but statistically significant decrease in heart rate was evident following 6 months of inorganic nitrate intervention compared to no change in the placebo treated patients (see Supplementary Appendix Figure S4C). Furthermore, these differences in BP were not related to any significant differences in anti-hypertension medications between the two groups at 6 months (Supplementary Appendix, Table S9).

**Table 6:** Adverse events by treatment allocation.

Adverse Event	Inorganic Nitrate (n = 150)	Placebo (n = 150)
**Total number of events (% male)**	**127 (85.1)**	**141 (92.9)**
**Cardiovascular**		
Abdominal aortic aneurysm repair	1	0
Admission with dizziness and sinus bradycardia	0	1
Admission to Hospital with atrial fibrillation	0	1
Angiogram and pressure wire study for medical management	4	0
Angiogram but no significant obstructive disease	2	2
Cerebrovascular accident	1	1
Distal wire perforation during index percutaneous coronary intervention	1	0
Hypotension requiring hospital admission	1	0
Left ventricular failure not requiring admission but A&E attendance	0	1
Light-headedness	1	0
Loss of consciousness and Bradycardia	1	0
New diagnosis of angina	10	12
Non-ST Elevation Myocardial Infarction	2	6
Palpitations	2	2
Percutaneous coronary intervention in other vessels	4	4
Peripheral Vascular Disease	2	1
Prolonged hospitalisation for left ventricular failure	1	0
Shortness of breath	12	10
Shortness of breath due to valve disease	1	0
ST Elevation Myocardial Infarction	0	2
Target vessel revascularisation	7	9
Tachy-Brady Syndrome	0	1
Troponin negative chest pain not requiring admission	1	3
Troponin negative chest pain requiring hospital admission	0	4
**Respiratory**		
Admission with lower respiratory tract infection	2	0
Community acquired pneumonia	1	0
Diagnosed and treated for Tuberculosis	0	1
Diagnosed with COPD	0	1
Exacerbation of asthma	1	0
Exacerbation with COPD required hospital admission	1	0
Exacerbation with COPD required ITU admission	1	0
Shortness of breath requiring hospital admission overnight	0	1
**Gastrointestinal**		
Abdominal Pain	0	2
Abnormal liver function tests	1	0
Anal Fissure	0	1
Autoimmune hepatitis	0	1
Change in bowel habit	1	0
Cholecystectomy	1	1
Cholecystitis	0	1
Constipation	1	0
Diarrhoea	4	3
Gallstones admitted to hospital	1	1
Gastrointestinal bleed associated with haemorrhoids	1	0
Gastrointestinal bleed associated with naproxen	1	0
Norovirus	1	0
Rectal bleeding	1	0
Rectal bleeding due to colitis	1	0
Rectal bleeding due to Polyp	0	1
Reflux Oesophagitis	1	0
Small bowel obstruction	0	1
Stomach Ulcers	0	1
Taste	7	5
Vomiting	5	2
**Neurological**		
Dizziness	1	3
Headache–went to A&E, normal CT Head	1	0
Headache	0	1
Memory Issues	0	1
Paraesthesia in hands and arms (right side)	1	0
Vertigo	1	0
**Ophthalmology**		
Diabetic retinopathy	1	0
Subconjunctival haemorrhage	0	1
Uveitis	0	1
Cataract surgery left eye	1	0
**Endocrine**		
Raised sugars requiring increase in dose of diabetic medications	2	2
**Musculoskeletal**		
Back pain	2	3
Bilateral calf pain	1	0
Bilateral leg cramps	0	1
Broken Achilles tendon rupture requiring surgery	1	0
Broken ankle (mechanical–requiring surgery to ankle)	0	1
Broken left arm	1	0
Charcot's foot (left)	0	1
Diagnosed with rheumatoid arthritis	1	1
Frozen Shoulder	0	1
Hip Pain	1	0
Knee replacement surgery	0	0
Left knee pain	1	1
Lower limb pain	0	2
Neck pain	1	0
Non-Cardiac Chest Pain	6	8
Right side chest pain following mechanical fall	1	0
Right upper limb pain	0	2
Sciatica	1	0
Shoulder pain	0	1
**Genitourinary Tract**		
Benign prostatic hypertrophy	0	2
Haematuria, diagnosis of renal staghorn calculi	0	1
Haematuria–no cause found	1	0
Lithotripsy for renal stones	0	1
Peyroine's disease	0	1
Urinary Tract Infection	2	1
**Dermatological**		
Back abscess	0	1
Drain abscess	0	1
Excision of left lower eye lid cyst	1	0
Right lower leg cellulitis	0	1
Umbilical hernia repair	1	0
Wound on palm of hand requiring dressing and cleaning	0	1
**Malignancy**		
Blader cancer	0	1
Bowel cancer	0	1
Lung cancer	0	2
Prostate cancer	0	1
**Haematological**		
Diagnosed with Polycythaemia Vera Exon	1	0
Leukaemia (non-life threatening)	0	1
**Other**		
Admission to hospital with fever	1	0
A&E admission with feeling anxiety	0	1
COVID-19 Infection	0	1
Death	3	6
Depression	1	0
Epistaxis	1	0
Hyperventilation	0	1
Mechanical Fall attendance to A&E	0	1
Middle ear infection	0	1
Non-specifically unwell requiring hospital admission	1	0
Tooth pain	1	0

### Once a day inorganic nitrate raises levels of nitrite and nitrate in blood, urine, and saliva in patients with CAD

There was a statistically significant increase at 6 months in the plasma nitrate concentration compared to baseline of ∼6.1—fold and a significant increase of ∼2.0–fold in plasma nitrite concentration. These rises were likewise accompanied by statistically significant increases of saliva nitrate concentration of approximately 5.6–fold and saliva nitrite of approximately 3.5–fold, as well as urine nitrate concentration of ∼5.0–fold and nitrite concentration of ∼3.3–fold (see Supplementary Appendix Table S8). In contrast the levels of nitrite or nitrate remained mostly stable over time in the placebo-treated group with only a small (1.3-fold) rise in plasma nitrate concentration and decrease (0.6-fold) in salivary nitrate at 6 months. Inorganic nitrate treatment caused a statistically significant, but clinically unimportant, rise in methaemoglobin levels from 0.3% to 0.5% (see Supplementary Appendix Figure S5). Post-hoc analysis of the dose received relative to body weight and comparison with extent of in-stent LLL in both groups identified a statistically significant (P = 0.0083) inverse correlation between dose received and extent of LLL (Supplementary Appendix Figure S6).

## Discussion

In NITRATE-OCT, a prospective double-blind, randomised placebo-controlled trial, in patients with chronic coronary syndrome undergoing PCI, a once-daily treatment of inorganic nitrate (using a dietary approach) for 6-months, on top of standard medical therapy, led to a statistically significant reduction of in-stent LLL, as measured by QCA, meeting the primary endpoint for the study, although the total number of patients fell short of the original sample size estimation (i.e., 209 versus 220 respectively). With this caveat this result demonstrates for the first time that a simple once-daily dietary inorganic nitrate treatment reduces ISR in patients undergoing PCI for stable angina.

The incidence of ISR and thrombosis, whilst substantially reduced by drug-eluting devices and anti-platelet therapy, still represent a significant cause of treatment failure and are associated with increased morbidity and mortality.^46^, ^47^, ^48^ Evidence published last year from the ISAR-DESIRE-3 trial, with the longest recorded follow-up to date, identified that most cardiac events (device-oriented composite endpoint) occur in the first year post intervention, but continue to occur over the following 10 years to 55–62%, with revascularisation occurring at a rate of 38.6–58%.^49^ Recent efforts to limit ISR have focussed upon use of bioresorbable stents, to overcome the issues of insufficient arterial healing. However, to date no benefits versus DES have been observed and, if anything, an increased risk of thrombosis and target lesion failure are evident in meta-analyses.^50^^,^^51^ Intracoronary brachytherapy continues to be used in a few selected centres globally with some success although the evidence base remains soft.^52^^,^^53^ A number of pharmacotherapeutic approaches have also been tested, in particular interventions aimed at targeting the immune response including oral rapamycin,^54^^,^^55^ i.v. systemic paclitaxel via nanoparticles,^56^ and oral prednisolone.^14^ However, these strategies use short courses of high-doses of these drugs and only small benefits with BMS use have been observed and, moreover, the outcome is still inferior when compared to early generation DES.^14^^,^^54^, ^55^, ^56^, ^57^ Thus, the unmet need remains, and this underlies the rationale for assessment of the utility of dietary inorganic nitrate to deliver, anti-inflammatory, anti-thrombotic and anti-proliferative NO.

QCA is a well-established method used in the clinical setting for assessing LLL and the efficacy of different PCI strategies.^58^^,^^59^ Our findings indicate that sustained dietary inorganic nitrate supplementation reduces LLL. Moreover, the reduction in LLL was associated with a ***trend*** towards reduced MACE, in the ITT analysis, that is apparent at 1-year follow-up and beyond, and statistically significant in the per-protocol analysis with exclusion of all patients who did not receive a stent. Whilst this observation should be interpreted with caution it does fit, nevertheless, with the observed reduction in LLL. An important point to note is that the mean standard deviation was lower than the assumed standard deviation, this is likely related to improvements in both stent technology and methodology of stent implantation over the study period and is also evident with lower standard deviation measurements in more recent studies.^60^

The OCT analyses were conducted to explore mechanisms of any effect seen but the study was not powered to see a difference in neointimal hyperplasia between the two groups. Importantly, it is noteworthy that OCT was conducted at 6 months only with no baseline measurements. Overall, the OCT analysis demonstrated that the vessel lumen area was numerically increased in the dietary inorganic nitrate group versus placebo group. This observation could be attributed to a general vasodilation of the artery or due to less restenosis. Whilst the decreased BP with dietary nitrate treatment supports the theory of vasodilation, the OCT measures demonstrating identical neointimal thickness between the groups, but significantly increased lumen volume, may suggest a relative reduction of neointimal formation. This data in part matches the QCA data supporting a reduced ISR with reduced in-stent and in-segment LLL. These observations also fit well with the reduced number of ‘late’ (>1 year) presentation of events in the inorganic nitrate-treated group. However, despite the improvements in QCA outcome measures this OCT data does not demonstrate a reduction in neointimal %, volume or area. The possibility that bias drives this absence of effect is largely excluded by the sensitivity analysis conducted. However, the Independent CoreLab also excluded some OCT scans due to technical failings in image quality and thus we cannot entirely exclude this possibility A further prospective investigation powered for OCT-derived outcomes is required to address this.

Whilst the MACE outcome was powered off a 6-month endpoint no difference in MACE between the groups at this timepoint was evident. However, our follow-up extended over two years and just prior to one-year the Kaplan–Meier survival curves crossed and further substantial separation occurred over the next year of follow-up. Further breakdown of the MACE events identified that this difference in MACE was driven by differences in death and MI occurring over time, and not related to events in the first year after intervention. However whether these events were specifically due to occlusion of the culprit artery is unknown. Whilst this observation should be interpreted with caution it does fit, nevertheless, with the observed reduction in LLL and previous studies linking MACE to ISR.^61^ One could speculate that chronic systemic inflammation leads to a progressive blocking of the artery that becomes symptomatic over a prolonged period, as evidenced by the MACE burden in the placebo arm over the second year of follow up. The impact of suppression of this low-grade systemic inflammation in the immediate period (6 months) following PCI with inorganic nitrate treatment thus would only become evident in MACE at later timepoints. Further mechanistic studies imaging the stented artery at 6 months, together with analysis of inflammatory markers may provide some indication of this possibility. However, since the MACE events crossed at one-year further studies conducting additional imaging at this later timepoint may be useful.

Analysis of the nitrate content of the juice intervention indicated that on average patients received 7.5 mmol/day versus 0.1 mmol/day in the placebo arm, equating to a daily dose in the treatment arm of 90 μmol/kg nitrate (5.5 mg/kg/day). Furthermore, there was an inverse relationship between the dose of nitrate and in-stent LLL (Supplementary Appendix Figure S6). In those receiving inorganic nitrate, the levels of nitrate and nitrite in saliva, plasma and urine increased, indicating a fully functioning non-canonical pathway for NO.^29^ This finding is important since atherosclerotic disease has been linked with an altered microbiome, particularly of the gut, that influences disease progression.^62^^,^^63^ Whether this might be the case for the oral microbiome is unknown and an issue that will be interrogated in future analysis of the microbiome samples collected in this trial. However, salivary nitrate and nitrite concentrations increased substantially and the ratio of salivary nitrate to nitrate in this study averaged at 0.3 which is typical for oral nitrate reduction and as shown in previous studies.^39^ It is of note that with the inorganic nitrate intervention the levels of the anions measured are similar to those observed in both healthy volunteers and patients with cardiovascular disease where efficacy has been demonstrated; the latter including hypertension^37^ and hypercholesterolemia.^39^ Confirmation of NO delivery, in addition to safety, is further demonstrated by the minor rise in non-symptomatic met-Hb levels and the decrease in BP. The former reflects the scavenging of free NO by oxy-Hb and provides confidence in the potential of dietary inorganic nitrate to deliver NO in the CAD setting. However, the very low levels support the view that dietary doses of inorganic nitrate that approximate to the ADI are safe, as described in assessments by the EU/WHO and discussed in recent reviews.^29^^,^^41^^,^^64^

It is possible that the decreased LLL and trend to decrease in MACE outcome is a consequence of the significant BP lowering effects of dietary inorganic nitrate. Indeed, large scale meta-analyses suggest that reductions in SBP, by standard BP-lowering agents, are closely associated with reductions in MI and stroke.^65^, ^66^, ^67^ However, data from the SPRINT study, which enrolled over 9000 patients with hypertension, whilst demonstrating benefit cast doubt over the BP/MI association since intensive reduction in BP was not associated with significant reduction in MI or cerebrovascular accident, but rather caused decreases in heart failure.^68^^,^^69^ Thus, the trend to a reduction in MACE may not relate directly to a reduction in BP but in combination with the decrease in LLL may lead to better long-term clinical outcomes; an issue that can be assessed in larger studies.

Despite the general prevailing view that NO-signalling exerts a prohibitive influence over atherosclerotic, pro-inflammatory and pro-proliferative^70^, ^71^, ^72^ pathways, clinical trials assessing the impact of sustained NO delivery upon ISR and thrombosis are scant. In the landmark ISIS-4^73^ trial chronic use of ***organic nitrate*** (glyceryl trinitrate-GTN) in patients post–MI did not improve MACE. Exactly why no benefit was evident is uncertain but it may relate to insufficient elevations of circulating NO concentrations, the short duration of treatment (4 weeks) or possibly to the known tachyphylaxis of response to organic nitrate with continued use (although a low dose thought to induce less tachyphylaxis was used specifically to avoid this complication).^74^ This latter effect is of relevance to NITRATE-OCT. A key difference between organic nitrates and inorganic nitrate/nitrite, in terms of delivery of NO, is that the latter do not suffer tachyphylaxis with continued use.^33^^,^^37^^,^^75^ This difference highlights one of the key advantages of inorganic nitrate over organic nitrate as a potential therapeutic.

In this study, due to operator decision making during the procedure, only 271 of the consented and randomised 300 patients received a DES. Moreover, only 208 angiograms were available to analyse and thus ∼77% of the consented patients treated with a stent. Our percent of patients receiving a stent for which primary outcome data was available is slightly lower than that achieved in some other studies of similar recruitment targets (e.g., the OSIRIS trial^15^ and the NIREUS study^76^). Importantly, NITRATE-OCT was conducted during, and thus impacted by, the COVID-19 pandemic due to an inability to recall all the patients for their planned repeat angiogram. Our sensitivity analyses suggested that the characteristics of the patients lost to follow-up were similar between the two treatment arms. This observation suggests that the positive outcome in this study is unlikely related to a bias associated with dropout. However, it is noteworthy that other recent trials in similar patient cohorts have reported similar (e.g., 28% at 9 months)^77^ and even greater (∼45% at 6 months)^78^ loss to follow-up than in this study. Further assessment of the causes of this loss, to potentially determine any mitigating actions to prevent this, would be useful for future trial design. It is also important to note that the power calculation for the primary endpoint, which was LLL using QCA, required 220 patients in total. Although we had only a 69% re-angiogram rate, there were 208 patients that were included in the final ITT analysis. Therefore, the study failed to recruit to the estimated sample size required.

The statisticially significant difference in the primary outcome finding whilst positive should be viewed with caution due to failure to reach the sample size estimate. Although, irrespective of whether angiographic follow-up occurred, all patients remained in the powered MACE outcome analysis. Our dataset, suitable for analysis, was reduced further due to technical quality issues with the imaging, 20 patients and 21 vessels were excluded from the final analysis by the Independent Core Lab. Interestingly, if all angiograms were included in the final dataset, then a significant difference between the two groups in terms of LLL is still observed (Table 3).

This phase II was a single centre study, and this represents an important limitation. Whilst the community served by the Barts Heart Centre is one of the most diverse and underrepresented in the UK drawing patients from a catchment area of 3 million, it will be important to demonstrate generalisability of the findings in a follow-on multi-centre phase III trial powered for MACE. Additionally, in this study the proportion of female patients in the cohort was only 13%. Thus, to ensure the generalisability of any findings to both sexes, a study design seeking to increase the proportion of females recruited needs to be employed, although currently the best options for this remain uncertain for a trial where consecutive presenting patients are recruited to minimise bias. This is an issue that has been acknowledged recently in numerous high-profile calls to action to redress the paucity of assessment of outcomes in women.^79^

Finally, intravascular imaging was not mandated at the index procedure, to better simulate the ‘real-world’ population at the time. Indeed, currently intravascular rates are less than 15% within most cardiac services.^80^ Intravascular imaging could have led to improved ‘baseline’’ stent optimisation, which may have impacted the overall results with a reduction in LLL across both groups.

The results of this Phase II clinical trial demonstrate that a daily oral ingestion of inorganic nitrate is a safe and simple method that can be utilised to provide a sustained elevation of circulating nitrite and thus NO that is associated with a potential reduction in LLL in patients undergoing drug eluting stent insertion.

## Contributors

K.S. Rathod∗: data curation, formal analysis, funding acquisition, data interpretation, writing—original draft and takes responsibility for the data and checked and verified the underlying data.

A. Mathur∗: conceptualisation, funding acquisition, investigation, methodology, data interpretation, writing—original draft and takes responsibility for the data.

A. Shabbir: data collection, writing—original draft.

R.S. Khambata, writing– review & editing.

C. Lau: data collection, writing– review & editing.

AM. Beirne: data collection, writing– review & editing.

I. Chhetri: project administration.

M. Ono: project administration.

D.R. Belgaid: data collection, G Massimo: data collection, A. Ramasamy: data collection, V. Tufaro: data collection, A.K. Jain: data collection, N. Poulter: methodology, study design.

E Falaschetti: methodology, study design.

D.A. Jones: data collection.

H.M. Garcia–Garcia: formal analysis and takes responsibility for the QCA and OCT data.

C. Bourantas: formal analysis.

A. Learoyd: formal analysis.

H. R. Warren: formal analysis.

A. Ahluwalia: conceptualisation, funding acquisition, investigation, methodology, data interpretation, writing—original draft and takes responsibility for the data and checked and verified the underlying data.

∗ Both authors had equal contributions.

All authors read and approved the final version of the manuscript.

## Data sharing statement

De-identified patient data will be available upon reasonable request to the corresponding author (via email) after institutional approval and with a signed data access agreement, with no time limits after publication.

## Declaration of interests

Amrita Ahluwalia is a Co-Director of Heartbeet Ltd.

Hector M. Garcia–Garcia has received speaker's fee from Biotronik, Boston Scientific, Medis and support for attending meetings from Biotronik, Boston Scientific.

All remaining authors declare no competing interests.

## Appendix A Supplementary data

Summary Table

NITRATE-OCT Protocol Paper

SAP- Nitrate OCT version 0.2 August 2019
