# Supplementary figures and images for: The NITRATE-OCT study-inorganic nitrate reduces in-stent restenosis in patients with stable coronary artery disease: a double-blind, randomised controlled trial
Source: eClinicalMedicine. 2024 Oct 18;77:102885. doi: 10.1016/j.eclinm.2024.102885 (PMC11513660; doi:10.1016/j.eclinm.2024.102885)

**Summary Table of Approvals for the NITRATE-OCT Study**


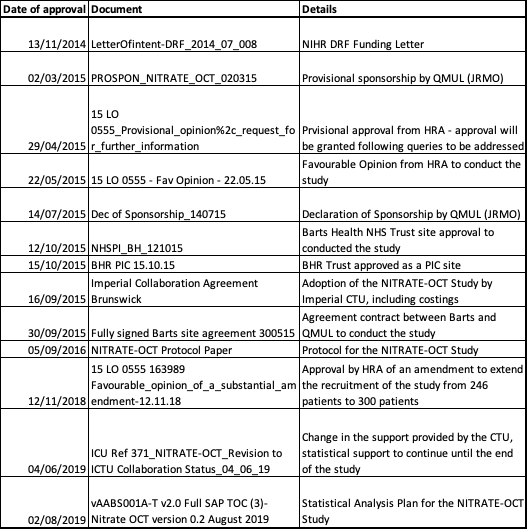

Supplement: Summary Table [file mmc1.docx]
