# Supplementary material for: The NITRATE-OCT study-inorganic nitrate reduces in-stent restenosis in patients with stable coronary artery disease: a double-blind, randomised controlled trial
Source: eClinicalMedicine. 2024 Oct 18;77:102885. doi: 10.1016/j.eclinm.2024.102885 (PMC11513660; doi:10.1016/j.eclinm.2024.102885)
Supplement: SAP- Nitrate OCT version 0.2 August 2019 [file mmc3.pdf]

Statistical Analysis Plan (SAP)

NITRATE-OCT

A randomised, double-blind, placebo-controlled study investigating the effects of dietary nitrate on vascular function, platelet reactivity and restenosis in stable angina

Protocol Version 3.0

Study Investigators:

Amrita Ahluwalia, Anthony Mathur, Mark Caulfield, Neil Poulter.

SAP Working Group:

Jackie Cooper, Krishnaraj Rathod, Emanuela Falaschetti.

Document version history log:

Version	Date	Author	Summary of changes made

Nitrate OCT. Protocol Version 3.0

1. Approval Signatures

2. Content

1. Approval Signatures 2
2. Content 2
3. Abbreviations 4
4. Introduction/Study summary 5
5. Study Objectives / Hypotheses Testing 5
5.1. Primary Objectives 5
5.2. Secondary Objectives 5
6. Design 5
6.1. Study Design 5
6.2. Treatment Groups 5
6.3. Study Population 6
6.4. Eligibility Criteria 6
6.5. Blinding 6
6.6. Sample Size 6
6.7. Schedule of Time and Events 6
6.7.1. Schedule of Assessment 7
6.8. Randomization 8
7. Populations of Analysis Sets 8
7.1. Intent-to-Treat/randomised Population 8
7.2. Safety population 8
7.3. Per protocol Population 8
8. Variables of Analysis 8
8.1. Primary Efficacy Variable 8
8.2. Secondary Efficacy Variables 8
8.3. Safety Variables 9
8.4. Demographic Variables 9
9. Statistical Methodology 9
9.1. General Methodology 9
9.1.1. Types of analysis 9

9.1.2. Levels of significance 10
9.1.3. Covariates 10
9.1.4. Missing data conventions 10
9.1.5. Model assumptions 10
9.2. Patient Flow (CONSORT diagram) 11
9.3. Baseline Demographics 11
9.4. Compliance to study drug 11
9.5. Primary Efficacy Analysis 11
9.6. Secondary Efficacy Analysis 11
9.6.1. Endothelial function 11
9.6.2. TVR/MACE 11
9.6.3. In-segment late loss 12
9.6.4. Plaque size 12
9.6.5. Inflammatory markers/ platelet activation 12
9.7. Safety Analysis 12
9.7.1. Adverse events (AE) 12
9.7.2. Deaths or serious adverse events (SAE) 12
9.8. Interim Analysis 13
9.9. Sensitivity Analysis 13
9.10. Subgroup Analysis 13
9.11. Tables to present 13
9.12. Figures to present 16
10. Amendments to Version 1.0 16
11. REFERENCES 17
12. Appendix 18
12.1. CONSORT 2010 Flow Diagram 18
12.2. Table of demographic and procedural characteristics 19

3. Abbreviations

ACS	Acute Coronary Syndrome
AE	Adverse Event
ADP	Adenosine Diphosphate
BMS	Bare Metal Stents
CI	Confidence Interval
CRISP	Cardiac Remote Ischemic Preconditioning in Coronary Stenting
CVA	Cerebrovascular Accident
DES	Drug Eluting Stents
DSMB	Data Safety Monitoring Board
FMD	Flow Mediated Dilatation
hsCRP	High sensitivity C-Reactive Protein.
IL-6	Interleukin6
IQR	Interquartile Range
ISLL	In-stent Late Loss
MACE	Major Adverse Cardiac Events
MAR	Missing At Random
MCAR	Missing Completely At Random
MI	Myocardial Infarction
MNAR	Missing Not At Random
MLD	Minimum Luminal Diameter
NO	Nitric Oxide
OCT	Optical Coherance Tomography
PCI	Percutaneous Coronary Intervention
PDE	Phosphodiesterase
PI	Principal Investigator
PMM	Pattern Mixture Model
PWA	Pulse Wave Analysis
PWV	Pulse Wave Velocity
QQ	Quantile-Quantile

SAE Serious Adverse Event

SD Standard Deviation

TVR Target Vessel Revascularisation

XO Xanthine Oxidase

4. Introduction/Study summary

The opening of coronary arteries via PCI with stent insertion in coronary artery disease, including stable angina, is the treatment of choice to protect against future cardiac events. A major determinant of prognosis after treatment is the reocclusion of the affected arteries. A number of specific phenomena have been linked with reocclusion including persistent endothelial dysfunction, increased platelet reactivity and restenosis. Therefore, strategies that might limit or correct these phenomena have clear therapeutic potential. As recent evidence suggests that dietary nitrate has a range of biological effects in the body that may reduce restenosis, this study will assess whether dietary nitrate in addition to conventional therapy in stable angina patients might, through elevating circulating nitrite levels, exert anti-platelet effects, improvement of endothelial function and improvements of intimal hyperplasia resulting in possible reductions of restenosis rates post PCI and stent implantation.

5. Study Objectives / Hypotheses Testing

5.1. Primary Objectives

The primary analysis will test whether dietary nitrate improves intimal hyperplasia post PCI and stent implantation.

5.2. Secondary Objectives

Secondary objectives will determine whether dietary nitrate ingestion elevates circulating nitrite, exerts anti-platelet effects or improvement of endothelial function and the mechanisms involved in this effect. We will also determine whether restenosis rates and MACE outcomes differ by treatment group.

6. Design

6.1. Study Design

This is a single-centre double- blind randomised placebo controlled trial. Patients are randomised in a 1:1 ratio (using an on-line randomisation database) to receive 70 ml of a beetroot juice concentrate containing 5 mmol nitrate or nitrate-depleted placebo juice concentrate (placebo). Block randomisation is used with patients stratified into diabetics and non-diabetics to ensure balance of treatments within these groups. The study aims to recruit 230 patients after drop-out.

6.2. Treatment Groups

Patients will be randomised to receive 70 ml of beetroot juice or nitrate free placebo juice (James White Drinks). The juice used enables reliable dosing of approximately 5 mmol nitrate, a dose that we have

previously shown to be effective in reducing blood pressure, and improving endothelial and platelet function.

6.3. Study Population

Patients will be recruited at Barts Health NHS trust within the Barts and The London Heart Attack Centre, based at The Barts Heart Centre, St. Bartholomew's Hospital. Patients will be aged at least 18 years with stable angina and undergoing elective PCI.

6.4. Eligibility Criteria

Eligible patients will have stable angina diagnosed by a cardiologist, be on optimal medical therapy and undergoing angioplasty to treat residual symptoms. A full list of inclusion and exclusion criteria are given in the protocol (sections 6.2, 6.3)

6.5. Blinding

Assignment to active or placebo will remain blinded until data lock and statistical analysis at the end of the study. If un-blinding is required for clinical reasons the PI or clinical research fellow will be informed. A list of the unblinded treatments will be kept in a secure location at the William Harvey Heart Centre. The un-blinding procedure will be available at all times (24hours day/7 days a week).

6.6. Sample Size

The study has been powered for both primary and secondary endpoints. The largest sample size is needed for the secondary endpoint of MACE. The CRISP Stent Trial (1) found a reduction in MACE from 12.5% to 3.6% in stable angina patients for a group receiving remote ischemic pre-conditioning compared to a control group. For a one-tailed test a total number of N=230 would be needed to detect an effect of this size with 80% power at the 5% significance level. The sample size has been increased by 30% to account for drop-out or withdrawal/non-compliance giving a final sample size of N=300.

For the primary endpoint, the average means and SDs from 22 trials measuring late loss have been calculated as mean 1.27mm and SD 0.55 (2). The sample size of 230 after withdrawal will give 80% power to detect a difference of 0.205 (<50% of the effect size seen with PDE inhibition) at the 5% level using a two-sided test.

Our in-house FMD data suggests increases of FMD of 1% with an SD of 1.5%. Assuming no change in the control group the study will have >99% power to detect an effect of this size.

For inflammation markers, NO biochemistry and platelet aggregation, using alpha=0.025 to allow correction for multiple comparisons at two time points the study has 80% power to detect a difference of 0.3 standard deviations. In our most recent clinical study in hypercholesterolemics once daily dietary nitrate for 6 weeks resulted in a decrease in % platelet monocyte aggregates of -7.6 compared to an increase of 10.1 in the placebo arm (SD=21.5), and our study would have >99.9% power to detect an effect of this size.

6.7. Schedule of Time and Events

Details of recruitment, screening and assessments are given in the protocol (section 7). Patients will be invited to participate by means of posters and advertisements directed at patients suitable for this study and participants who contact the main research team will be invited to attend the William Harvey Clinical Trials Centre one week later where they will have the opportunity to ask any questions and make a decision regarding participation.

Participants will attend for a screening visit (visit one) within 60 days, prior to commencing the treatment. Visit 2 will be a week ± 2 days before their scheduled angioplasty. Recruits will then be randomised. Treatment will begin a day before the scheduled angioplasty and will continue for 6 months. Patients will record their daily juice taking in a diary. Participants will then be asked to come back at 6 months (visit 3) and 12 months (visit 5) to repeat all tests.

Participants will be scheduled for a second angiogram with OCT at 6 months ± 1 month (visit 4). After 2 years, the participant will be contacted by telephone for assessment of MACE. The study will end after 1 year after the telephone follow-up of the last patients and analysis will take place.

6.7.1. Schedule of Assessment

Procedures	Screening Visit 1	Visit 2 Baseline (Prior to PCI)	During PCI	Visit 3 6 months post PCI	Visit 4Angiogram and OCT 6 months ± 1 month post PCI	Visit 5 12 months post PCI	2 years
Screening for inclusion /exclusion criteria	x						
Physical exam		x					
Consent		x					
Randomisation		x					
Bloods		x		x		x	
Saliva		x		x		x	
Urine		x		x		x	
PWA and PWV		x		x		x	
Flow mediated dilatation		x		x			
Ingestion of juice (Nitrate replete or nitrate deplete placebo) + completing a daily juice diary		x	x	x			
PCI			x				
Angiogram and OCT					x		

**MACE follow-up**				x		x	
**Assessment for adverse events and reactions**		x	x	x	x	x	
**Assessment for adverse events and reactions via telephone call**							x

6.8. Randomization

Patients are randomised in a 1:1 ratio (using an on-line randomisation database) to receive 70 ml of a beetroot juice concentrate containing 5 mmol nitrate or nitrate-depleted placebo juice concentrate (placebo). Block randomisation is used with patients stratified into diabetics and non-diabetics to ensure balance of treatments within these groups.

7. Populations of Analysis Sets

7.1. Intent-to-Treat/randomised Population

For the efficacy analysis patients will be analysed according to the group they were assigned to, regardless of whether the treatment was received or whether the protocol was adhered to.

7.2. Safety population

The safety population will consist of all randomised patients who received at least one treatment dose analysed according to the actual treatment received.

7.3. Per protocol Population

The per protocol population will consist of patients who report continued use of the juice throughout the study and have no other reported protocol violations as determined prior to unblinding of the study.

8. Variables of Analysis

8.1. Primary Efficacy Variable

The primary endpoint will be in-stent late loss (ISLL) where late loss is defined as the MLD within the segment immediately post stent placement minus the MLD at 6 months.

8.2. Secondary Efficacy Variables

Secondary endpoints are:

1. Change in Endothelial function.

Endothelial function of the brachial artery in subjects will be assessed by FMD. FMD is a non-invasive method of assessing endothelial function in vivo. It utilises vascular ultrasound to measure the increase in the diameter of the brachial artery in response to increased flow (3) and will be conducted according to published guidelines (4). Change in endothelial function will be calculated as the 6 month FMD minus the FMD at baseline (visit 2).

2. TVR.

TVR rate is defined as the proportion of patients with repeat PCI in the target vessel, assessed at 6 months, 12 months and 24 months.

3. MACE

MACE rate is defined as the proportion of patients with MI, death, CVA or TVR assessed at 6, 12 and 24 months

4. Restenosis.

Restenosis rate is defined as the proportion of patients with percent diameter stenosis>50 at 6 months.

5. In-segment late loss

In-segment late loss is calculated as the MLD within the segment immediately post stent placement minus the within segment MLD at 6 months.

6. Plaque size

Plaque size will be measured using OCT at 6 months (visit 4).

7. Inflammatory markers

Inflammatory markers plasma and erythrocyte nitrite reductase and XO activity, hsCRP and IL-6 will be analysed. Changes in inflammatory markers will be calculated by subtracting the baseline level from the marker level at 6 months and 12 months.

8. Platelet activation

Platelet activation (P-Selectin and platelet-monocyte aggregates) and platelet aggregation (ADP, collagen, arachidonic acid) will be analysed. Changes will be calculated by subtracting the baseline level from the marker level at 6 months and 12 months.

9. Rise and changes in nitrate and nitrite levels

Increases in nitrate and nitrite levels will be analysed. Changes in nitrate and nitrite levels will be calculated by subtracting the baseline level from the level at 6 months.

8.3. Safety Variables

Safety variables will be AEs and SAEs.

8.4. Demographic Variables

Demographic, baseline and procedural variables to be included in the baseline characteristics table are listed in the table in the appendix (section 12.2).

9. Statistical Methodology

9.1. General Methodology

9.1.1. Types of analysis

The primary efficacy analyses will be on an intention to treat basis with patients analysed according to the group they were assigned to, regardless of whether the treatment was received or whether the protocol was adhered to.

Efficacy analysis will also be performed on the per protocol population described in section 7.3.

9.1.2. Levels of significance

For the MACE endpoint, hypothesis testing will be carried out at the 5% (1-sided) significance level as specified in the protocol. Confidence intervals presented will be 95%, 1-sided.

For all other endpoints, hypothesis testing will be carried out at the 5% (2-sided) significance level. Confidence intervals presented will be 95% and 2-sided. For inflammatory markers p values will be Bonferroni corrected (p*2) to allow for analysis at individual time-points as specified in the protocol.

9.1.3. Covariates

The main analysis will adjust for diabetes as this is a stratification variable which should be accounted for in the analysis (5).

Adjustment for important baseline covariates is advised, as this can correct for any chance imbalances between randomisation groups and also improve the precision of the treatment effect estimates (5). The diabetes adjusted analysis will be considered as primary, with fully adjusted results including the additional covariates listed below also presented to support the conclusions.

For continuous variables where the outcome is change, baseline levels will be included as covariates in the models as described in sections9.5 and 9.6.

Covariates for inclusion are: Diabetes, previous bypass surgery (6) and procedural characteristics (culprit vessel, access site, DES use, number of stents, total stent length, mean stent diameter and procedural success).

9.1.4. Missing data conventions

The numbers missing for each outcome variable and covariate will be shown by treatment group, and a missing values patterns chart will be produced.

Missing covariates: If covariate data is missing the patient will be included in the analysis with an imputed value of the mean of the observed values and a missing-indicator variable as an additional covariate. This will give an unbiased estimate for the randomised treatment effect on outcome (7). As including a missing indicator for variables with only one missing value amounts to dropping the observation, missing indicator analysis will only be used if there are 3 or more missing values.

Missing outcomes:

For the longitudinal data, the mixed effects approach will provide an unbiased estimate of the treatment effect assuming the data is MAR. As MNAR data is difficult to rule out, we will conduct a sensitivity analysis using a pattern mixture model (PMM) approach, fitting models for the final time-point to assess any overestimation of the treatment effect due to missing outcome data(8).

For outcomes at a single time point we will first analyse the data using those with complete outcome data. Sensitivity analysis using a pattern mixture model (PMM) approach will be undertaken to assess the robustness of the results to departures from the MCAR assumption (8).

9.1.5. Model assumptions

For continuous outcome variables and covariates, normality of the distributions will be assessed using qq plots and histograms during blind review of the data and variables will be log-transformed if necessary to obtain a normal distribution prior to analysis.

If a normal distribution is not obtained for any outcome variable, p values will be obtained using the Mann-Whitney U-test instead of the t-test for the unadjusted analysis.

Residual plots will be used to assess the normality of residuals and homogeneity of variances and to identify outliers for the multiple regression models. If model assumptions are violated, sensitivity analysis will be conducted using a robust model.

For the logistic regression models, deviance residual plots will be used to identify outliers. To identify influential points the Pregibon $\Delta\beta$measure and the $\Delta\chi 2$ influence statistic will be plotted against predicted probabilities (9). If influential points are identified sensitivity analysis will be undertaken (removing these points from the model).

9.2. Patient Flow (CONSORT diagram)

Numbers of patients screened, excluded prior to randomisation by reason and overall, randomised and completing each phase of the study will be shown, following the Consort structure (Appendix 12.1).

9.3. Baseline Demographics

Demographic and baseline data will be summarised by randomisation group and overall. For continuous data the number of subjects, mean, SD, median and interquartile range will be shown. For categorical data number and percentage will be shown. No formal testing will be performed for the comparison of baseline characteristics.

9.4. Compliance to study drug

Compliance to the study drug will be assessed by number of remaining bottles, number of days capsules taken and number of doses missed. The number and proportion completing the course will be reported by treatment group.

9.5. Primary Efficacy Analysis

Mean (SD) and median [IQR] for in-stent late loss at 6 months will be presented by treatment group.. The diabetes adjusted and fully adjusted difference between treatment groups will be obtained from analysis of covariance models and presented with 95% confidence intervals. Baseline MLD will be included as a covariate along with the covariates described in section 9.1.3.

9.6. Secondary Efficacy Analysis

9.6.1. Endothelial function

Mean (SD) and median [IQR] for change in FMD will be presented by treatment group. Effect sizes (95% CI) will be obtained using multiple linear regression, with change in FMD at 6 months as the dependent variable and covariates as described in section 9.1.3.

9.6.2. TVR/MACE

The number and proportion of patients with TVR and MACE endpoints will be presented by treatment group at 6, 12 and 24 months. Denominators will be the number of patients with outcome data in each group at each follow-up and these numbers will be given in the results table. An overall odd ratios (95% CI) and p value for treatment will be obtained using mixed effects logistic regression. Patient will be fitted as a random intercept term and treatment and time point as fixed effects. Odds ratios and 95% confidence intervals at each time point will also be obtained after fitting of a time by treatment interaction in the model. P values and confidence intervals for the MACE endpoint will be one-sided as the study has been

powered for a one-tailed analysis which assumes a reduction in MACE for the active treatment group. Covariates will be included as described in section 9.1.3.

9.6.3. Restenosis

The number and proportion of patients with restenosis by treatment group at 6 months will be presented. The denominators will be the number of patients with restenosis outcome in each group at 6 months. Treatment effects will be presented as odds ratios (95% CI) obtained from logistic regression models.

9.6.4. In-segment late loss

Methods for the analysis of in-segment late loss will be the same as those used for the in-stent late loss (section 9.5).

9.6.5. Plaque size

Mean (SD) and median [IQR] for plaque size will be presented by treatment group. Effect sizes will be obtained using multiple linear regression.

9.6.6. Inflammatory markers/ platelet activation

Mean changes (SDs) will be presented for each marker by treatment group at 6 and 12 months. A mixed effects model will be fitted using maximum likelihood estimation. Patient will be fitted as a random intercept term and treatment and time point as fixed effects. Effect size (SE) and p value will be obtained for the overall treatment effect. A treatment by time interaction term will then be added to the model and coefficients will be obtained for the treatment effect at each time point. Bonferroni corrected p values for the treatment effect at individual time points will be obtained along with effect size and SE.

9.6.7. Nitrate/Nitrite levels

Mean changes (SDs) will be presented by treatment group at 6 months. Effect sizes will be obtained using multiple linear regression.

9.7. Safety Analysis

9.7.1. Adverse events (AE)

AEs will be summarised using counts and percentages. The number of subjects having at least one AE will be presented overall and tabulated by treatment. The number of subjects with AEs of mild/moderate/severe intensity will be shown overall and by treatment using the maximum severity experienced for each patient. The total number of AEs for each treatment, allowing multiple events per patient, will also be presented.

9.7.2. Deaths or serious adverse events (SAE)

SAEs will be listed separately along with details of the treatment and whether the event is unexpected and whether it is thought to be related to the treatment.

9.8. Interim Analysis

No formal interim analysis for efficacy is planned. Withdrawal rates and safety analysis as detailed in section 9.7 including reporting of adverse events will be undertaken for review by the DSMB and at the end of the trial. Other informal analysis may be undertaken at the request of the DSMB.

9.9. Sensitivity Analysis

Sensitivity analysis will be undertaken to assess any effect of informatively missing outcome data as described in section 9.1.4.

Sensitivity analysis will also be undertaken if necessary using robust models or excluding any outliers or influential points as described in section 9.1.5.

Sensitivity analysis will include per protocol analysis of the efficacy endpoints.

9.10. Subgroup Analysis

Pre-specified subgroup analysis will be performed for primary and secondary outcomes stratified by:

- organic nitrate use as part of routine therapy (Yes/no).
- DES vs. BMS

Diabetes adjusted effect sizes will be shown by subgroup and for all patients using the modified forest plot described by Cuzik (10). Interaction p values will also be shown.

9.11. Tables to present

- Table 1: Table of demographic and baseline characteristics, overall and by treatment group

- Table 2: Analysis of the primary endpoint.

	Treatment group		Diabetes adjusted		Fully adjusted	
**6 month:**	**Placebo**<br>**N=**	**Active**<br>**N=**	**b (95% CI)**	**P value**	**b (95% CI)**	**P value**
**ISLL**	Mean (SD)<br>Median [IQR]	Mean (SD)<br>Median [IQR]				

- Table 3: Endothelial function

	Treatment group		Diabetes adjusted		Fully adjusted	
**6 month:**	**Placebo**<br>**N=**	**Active**<br>**N=**	**b (95% CI)**	**P value**	**b (95% CI)**	**P value**
**Change in FMD**	Mean (SD)<br>Median [IQR]	Mean (SD)<br>Median [IQR]				

- Table 4: TVR/MACE

TVR or MACE endpoint	Time			Diabetes adjusted	Fully Adjusted
**Treatment group**	**6 months**	**12 months**	**24 months**	**Treatment Effect Active vs. placebo**	**Treatment Effect Active vs. placebo**
**Placebo**	n/N (%)	n/N (%)	n/N (%)	OR (95% CI) P=	OR (95% CI) P=
**Active**	n/N (%)	n/N (%)	n/N (%)		
**Unadjusted OR* (95% CI) P value**	OR (95% CI) P=	OR (95% CI) P=	OR (95% CI) P=		
**Fully adjusted OR* (95% CI) P value**	OR (95% CI) P=	OR (95% CI) P=	OR (95% CI) P=		

N=number with endpoint data at each time point within treatment group.
n=number with event occurring by specified time-point within treatment group.
*Treatment effect within each time-point

- Table 5: Restenosis

	Treatment group		Diabetes adjusted		Fully adjusted	
**6 month:**	**Placebo N=**	**Active N=**	**OR (95% CI)**	**P value**	**OR (95% CI)**	**P value**
**Restenosis**	n/N (%)	n/N (%)				

- Table 6: In segment late loss (as table 1).
- Table 7: Plaque size

	Treatment group		Diabetes adjusted		Fully adjusted	
**6 month:**	**Placebo**	**Active**	**b (95% CI)**	**P value**	**b (95% CI)**	**P value**

	N=	N=
**Plaque size**	Mean (SD)<br>Median [IQR]	Mean (SD)<br>Median [IQR]

- Table 8: Inflammatory markers/ platelet activation

**Marker name**	**Time**		**Diabetes adjusted**	**Fully adjusted**
**Treatment group**	**6 months**	**12 months**	**b* (95% CI)**<br>**Active vs. placebo**	**b* (95% CI)**<br>**Active vs. placebo**
**Placebo**	Mean (SD)<br>Median [IQR]	Mean (SD)<br>Median [IQR]	b (95% CI)<br>P=	b (95% CI)<br>P=
**Active**	Mean (SD)<br>Median [IQR]	Mean (SD)<br>Median [IQR]		
**Fully adjusted b** (95% CI)**<br>**P value**	b (95% CI)<br>P=	b (95% CI)<br>P=		

*beta coefficient for overall treatment effect
**beta coefficient for treatment effect at each time point.

- Table 9: Nitrate and nitrite levels.

	**Treatment group**		**Diabetes adjusted**		**Fully adjusted**	
**6 month:**	**Placebo**<br>**N=**	**Active**<br>**N=**	**b (95% CI)**	**P value**	**b (95% CI)**	**P value**
**Change in nitrate**	Mean (SD)<br>Median [IQR]	Mean (SD)<br>Median [IQR]				

- Table 3: Endothelial function

	**Treatment group**		**Unadjusted**		**Fully adjusted**	
**6 month:**	**Placebo**<br>**N=**	**Active**<br>**N=**	**b (95% CI)**	**P value**	**b (95% CI)**	**P value**
**Change in nitrite**	Mean (SD)<br>Median [IQR]	Mean (SD)<br>Median [IQR]				

- Table 9: Sensitivity analysis if model assumptions violated.
- Table 10: Safety analysis
- Table 11: Compliance to study drug
- Table 12: Per protocol analysis

9.12. Figures to present

- A Consort flow diagram will be presented (Appendix 12.1)
- Missing value plots
- Forest plots for subgroup analysis
- Sensitivity analysis for missing values

10. Amendments to Version 1.0

11. REFERENCES

1) Davies WR, Brown AJ, Watson W, McCormick LM, West NEJ, Dutka DP, et al. Remote Ischemic Preconditioning Improves Outcome at 6 Years After Elective Percutaneous Coronary Intervention: The CRISP Stent Trial Long-term Follow-up. Circulation: Cardiovascular Interventions. 2013;6(3):246-51.

2) Mauri L, Orav EJ, Kuntz RE. Late loss in lumen diameter and binary restenosis for drug-eluting stent comparison. Circulation. 2005;111(25):3435-42.

3) Celermajer DS, Sorensen KE, Gooch VM, Spiegelhalter DJ, Miller OI, Sullivan ID, et al. Non-invasive detection of endothelial dysfunction in children and adults at risk of atherosclerosis. Lancet. 1992;340(8828):1111-5.

4) Corretti MC, Anderson TJ, Benjamin EJ, Celermajer D, Charbonneau F, Creager MA, et al. Guidelines for the ultrasound assessment of endothelial-dependent flow-mediated vasodilation of the brachial artery: a report of the International Brachial Artery Reactivity Task Force. J Am Coll Cardiol. 2002;39(2):257-65.

5) International Conference on Harmonisation (ICH) Guideline E9 – Statistical Principles for Clinical Trials

6) Cassese S, Byrne RA, Tada T, Pinieck P, Joner M, Ibrahim T et al. Incidence and predictors of restenosis after coronary stenting in 10 0004 patients with surveillance angiography. Heart 2014; 100: 153-159

7) White IR and Thompson SG (2005). Adjusting for partially missing baseline measurements in randomised trials. Statistics in Medicine, **24,** 993-1007.

8) White IR, Horton NJ, Carpenter J. Pocock SJ. Strategy for intention to treat analysis in randomised trials with missing outcome data. BMJ 2011;342:d40

9) Hosmer DW, Lemeshow SA, Sturdivant RX (2013). Applied Logistic Regression. 3rd ed. Hoboken, NJ: Wiley

10) Cuzick J. Forest plots and the interpretation of subgroups. Lancet. 2005 Apr 9-15;365(9467):1308.

12. Appendix

12.1. CONSORT 2010 Flow Diagram

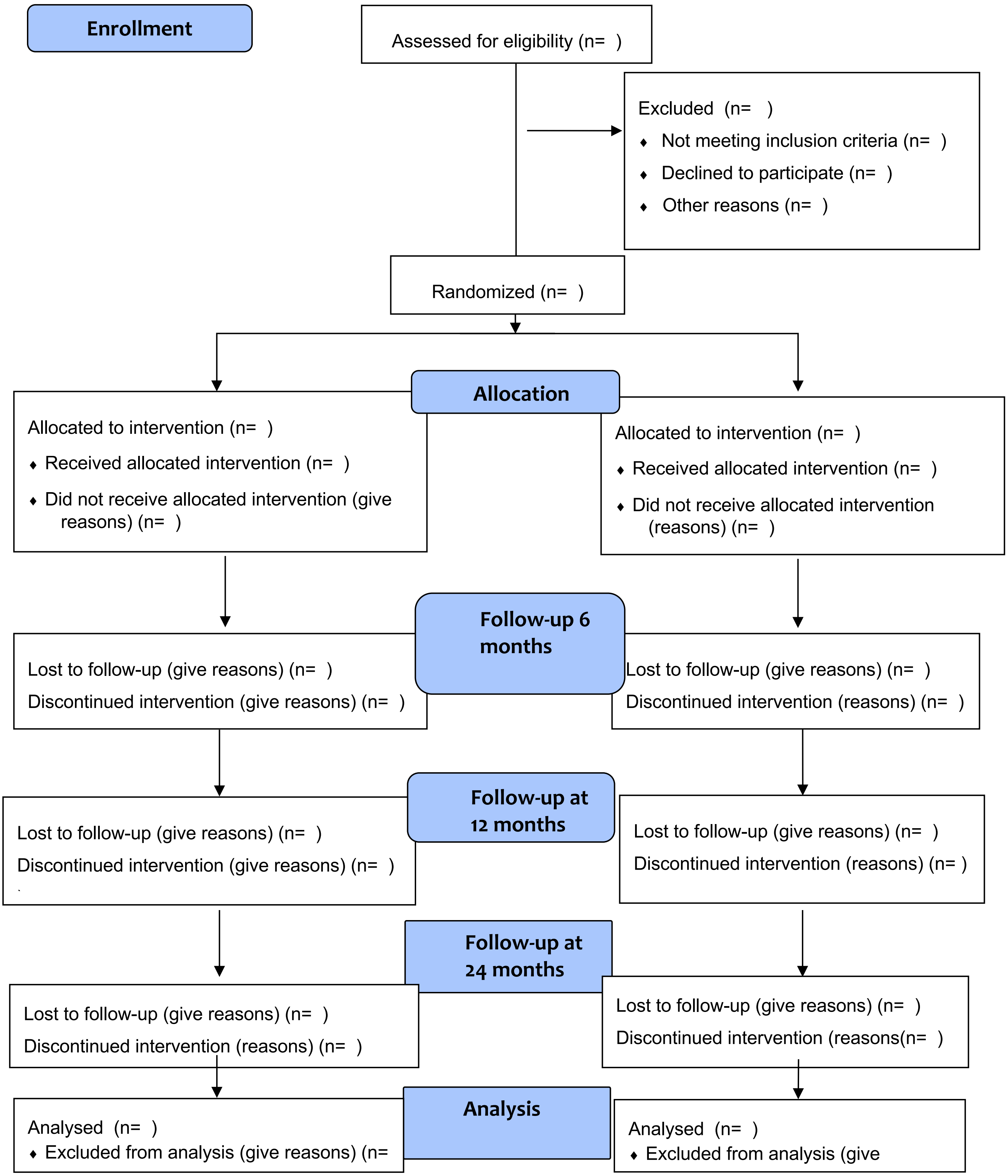

12.2. Table of demographic and procedural characteristics

Baseline demographics
Age (yr) (Mean±SD)
Sex (M/F)
Ethnicity
Caucasian
Afro-Caribbean
Asian
East Asian
Diabetes mellitus
Type I
Type II
Body-mass index (kg/m^2) (Mean±SD)[a]
Hypertension
Hypercholesterolaemia
Previous MI
Previous PCI
Previous CABG
Previous Smoker
PVD
CVA/TIA
NYHA
Class I
Class II
CCS
CCS I
CCS II
CCS III
Asthma
COPD
Previous History of CAD
Heart rate (BPM) (Mean±SD)

**Systolic BP (mmHg) (Mean±SD)**
**Diastolic BP (mmHg) (Mean±SD)**
**Culprit Vessel**
**Left main stem**
**Left anterior descending**
**Circumflex**
**Right coronary**

**Procedural characteristics**
Access site
Radial
Stenosis in non-culprit vessels
Left main stem
Left anterior descending
Circumflex
Right coronary
DES use
Number of stents used (Mean±SD)
Stent Type
Xience
Resolute Integrity
Promus Premier
Biofreedom
Stent length (Mean±SD)
First Stent
Second Stent
Third Stent
Fourth Stent
Stent diameter (Mean±SD)
First Stent
Second Stent
Third Stent
Fourth Stent
Treatment at time of PCI
Verapamil

Heparin
Heparin Dose (Mean±SD)
Aspirin
Clopiodgrel/Ticagrelor
Procedural Success
