# Supplementary material for: The NITRATE-OCT study-inorganic nitrate reduces in-stent restenosis in patients with stable coronary artery disease: a double-blind, randomised controlled trial
Source: eClinicalMedicine. 2024 Oct 18;77:102885. doi: 10.1016/j.eclinm.2024.102885 (PMC11513660; doi:10.1016/j.eclinm.2024.102885)
Supplement: Supplementary Figures and Tables [file mmc4.docx]

The NITRATE-OCT study - Inorganic nitrate reduces in-stent restenosis in patients with stable coronary artery disease: a double-blind, randomised controlled trial

**SUPPLEMENTARY RESULTS**

**Summary of patient withdrawal, dropout and adverse events**

All 300 patients recruited were discharged successfully following their procedures with no events during their stay. There were no differences between the groups in the length of hospital stay. There were 80 serious adverse events (SAE) (9 deaths – (1 following mitral valve surgery 5 month following the index procedure, 8 further deaths beyond 1 year from recruitment, which means they were 6 months following their end of the intervention), 19 re-hospitalisation for myocardial infarction (8 NSTEMIs and 2 STEMI), 1 patient admission for right sided chest pain following a mechanical fall, 7 patient admission with trop negative chest pain requiring admission, 1 prolonged hospitalisation for left ventricular failure, 14 patients underwent target vessel revascularisation, 9 patients with PCI but not target vessel revascularisation, 2 patients who had an angiogram and a pressure ire study but managed medically and 5 patients who had an angiogram but had unobstructed coronaries. There were 2 patents who were admitted with a stroke. There were 4 patients diagnosed with malignancy (1 with prostate cancer, 1 with bowel cancer, 1 with bladder cancer and 1 with lung cancer on chemo and radiotherapy). There was 1 patient admitted with a distal wire perforation during the index PCI, 1 patient admitted with troponin dizziness and sinus bradycardia. There were 2 patients who had a GI bleed (1 due to naproxen and 1 due to haemorrhoids). There were 3 patients admitted with gallstones and 1 subsequently had a callosectomy. There were 3 patients who were admitted with an exacerbation of chronic obstructive pulmonary disease (COPD) (1 required admission to the intensive care unit (ITU) admission), 1 patient with a lower respiratory tract infection. 1 patient had small bowel obstruction. 1 patient required lithotripsy for renal stones. There were 3 patients who required hospital admission for other reasons (1 for atrial fibrillation and 1 for shortness of breath). 1 patient had an abdominal aortic aneurysm repair. There have been 62 adverse events. Of these 27 patients had gastrointestinal upset resulting in 21 patients withdrawing from the study, 1 patient had light-headedness at 5.5 months into the study, 1 patient had light-headedness 3 months into the study, 1 patient had an unplanned diagnostic angiogram due to shortness of breath – angiogram was unremarkable, 1 patient was diagnosed with leukaemia but is not life-threatening, 1 patient was diagnosed with polycythaemia vera exon, 3 patients had troponin negative chest pain but not requiring admission to hospital, 4 patients had high blood sugars which was managed by increasing the doses of diabetic medications, 1 patient broke their ankle at work (mechanical in nature), requiring Achilles tendon surgery and 1 patient requiring a knee replacement following knee pain. There have been no suspected, unexpected serious adverse reactions. All 9 deaths in the study have been reviewed by the Chief Investigator and Principal Investigator of the study and who concluded that these events were not related to the intervention and therefore the study was not unblinded at the time.

A total of 54 patients had withdrawn before the 6 month angiogram. Of these 27 patients had withdrawn due to gastrointestinal upset, the remaining 27 patients had withdrawn as they did not want a second angiogram procedure. A further 11 patients were lost to follow up and 27 has no primary end-point due to not undergoing implantation with a stent at the time of the index procedure.

**Additional Clinical characteristics**

**Table S1.** Baseline medical therapy. Values shown as number (%) unless otherwise stated. Abbreviations: ACEi: Angiotensin-converting enzyme; ARB: Angiotensin receptor blocker. DOAC: direct oral anticoagulants, GTN; Glyceryl tri-nitrate.

|  | Inorganic Nitrate  (n=150) | Placebo  (n=150) |
| --- | --- | --- |
| **ACE inhibitor** | 89 (61.0%) | 69 (47.3%) |
| Ramipril | 65 (43.3%) | 61 (40.7%) |
| Enalapril | 3 (2.0%) | 0 (0%) |
| Perindopril | 6 (4.0%) | 2 (1.3%) |
| Lisinopril | 14 (9.3%) | 5 (3.3%) |
| Captopril | 1 (0.7%) | 1 (0.7%) |
|  |  |  |
| **ARB** | 20 (13.3%) | 26 (17.3%) |
| Lorsartan | 12 (8.0%) | 15 (10.0%) |
| Irbesartan | 3 (3.0%) | 1 (0.7%) |
| Candesartan | 5 (3.3%) | 9 (6.0%) |
| Telmisartan | 0 (0%) | 1 (0.7%) |
|  |  |  |
| **Diuretic** | 22 (14.7%) | 22 (14.7%) |
| Furosemide | 7 (4.7%) | 13 (8.7%) |
| Bendroflumethiazide | 4 (2.7%) | 4 (2.7%) |
| Spironolactone | 1 (0.7%) | 0 (0%) |
| Eplerenone | 3 (2.0%) | 4 (2.7%) |
| Indapamide | 7 (4.7%) | 4 (2.7%) |
|  |  |  |
| **Calcium Channel Blocker** |  |  |
| Amlodipine | 37 (24.7%) | 36 (24.0%) |
|  |  |  |
| **b Blocker** | 102 (60.0%) | 113 (75.3%) |
| Bisoprolol | 95 (63.3%) | 104 (69.3%) |
| Atenolol | 3 (2.0%) | 5 (3.3%) |
| Metoprolol | 1 (0.7%) | 3 (2.0%) |
|  |  |  |
| **Statins** | 137 (91.3%) | 137 (90.7%) |
| Atorvastatin | 120 (80.0%) | 124 (82.0%) |
| Pravastatin | 1 (0.7%) | 1 (0.7%) |
| Rosuvastatin | 11 (7.3%) | 9 (6.0%) |
| Simvastatin | 5 (3.3%) | 3 (2.0%) |

**Sensitivity analysis of the primary outcome analysis**

**Statistical analysis methods**

The main analysis was completed under the assumption that missing data in the primary outcome (in 92 patients) was missing at random. A pattern mixture model was implemented examining the robustness of this assumption in two linear regression models from Table 3. The difference between the mean LLL in missing patients (mean unobserved LLL) and the mean LLL in patients with this data (mean observed LLL) was set at a baseline of 0 (i.e. no difference in the mean outcome between those with missing outcomes and those with complete data) and varied between -0.5 and 0.5 at intervals of 0.1 for either both treatment groups, the placebo group only or the inorganic nitrate group only.

Baseline characteristics of patients with missing primary outcome data was compared to those patients included in the primary analysis using chi-squared tests for categorical variables with all calls >10, fisher’s exact test for categorical variables where any cell ≤10, and Student’s unpaired t-tests for continuous variables (all deemed to be normally distributed). These comparisons (Table S2) indicated that some baseline characteristics were related to missingness. To account for this scenario, a second pattern mixture model using the same parameters as indicated above was completed with the addition of the following auxiliary variables: age, peripheral vascular disease, and systolic blood pressure. 17 patients were missing systolic blood pressure data. The mean systolic blood pressure was imputed for these patients with a missingness indicator included as an additional covariate.

**Results**

The pattern mixture model set to the baseline difference between mean unobserved LLL and mean observed LLL of 0 for both treatment groups provided the expected results for the linear regression models: a difference between treatment groups of -0.16 (-0.25 to -0.06) for in-stent LLL and of -0.24 (-0.36 to -0.12) for in-segment LLL. This varied very little when the difference between mean unobserved LLL and mean observed LLL was adjusted in both groups (Table S3, Figure S1) suggesting that a difference of >-0.5 between mean unobserved LLL and mean observed LLL is necessary for the difference in in-stent LLL and in-segment LLL to become non-significant when comparing treatment groups.

When the difference between mean unobserved LLL and mean observed LLL was adjusted only in the placebo group, a negative difference between mean LLLs – resulting in a smaller mean LLL in the placebo group – led to a lack of significant difference between those on inorganic nitrate and those on placebo. For in-stent LLL a difference between mean unobserved LLL and mean observed LLL of -0.2 was sufficient to cause a lack of significant difference between treatment groups (-0.09 (-0.18 to 0.00)) (Table S3, Figure S1A). For in-segment LLL, a difference between mean unobserved LLL and mean observed LLL of -0.4 was necessary to cause a lack of significant difference between treatment groups (-0.11 (-0.23 to 0.01)) (Table S3, Figure S1B). A positive difference between mean LLLs – resulting in a larger mean LLL in the placebo group – by increased the estimated difference between treatment groups for both in-stent LLL (diff in mean LLL’s of 0.5: -0.32 (-0.42 to -0.22)) and in-segment LLL (diff in mean LLLs of 0.5: -0.40 (-0.53 to -0.28)) (Table S3, Figure S1).

When the difference between mean unobserved LLL and mean observed LLL was adjusted only in the inorganic nitrate group, a negative difference between mean LLLs – resulting in a smaller mean LLL in the inorganic nitrate group – increased the estimated difference in LLL between the two treatment groups for both in-stent LLL (diff in mean LLLs of -0.5: -0.30 (-0.40 to -0.20)) and in-segment LLL (diff in mean LLLs of -0.5: -0.38 (-0.51 to -0.26)) (Table S3, Figure S1). Increasing the difference between mean unobserved LLL and mean observed LLL – resulting in a larger mean LLL in the inorganic nitrate group – led to a lack of significant difference between treatment groups. For in-stent LLL a difference between unobserved mean LLL and observed LLL of 0.3 was sufficient to cause a lack of significant difference between treatment groups (-0.07 (-0.16 to 0.03)) (Table S3, Figure S1A). For in-segment LLL, a difference between unobserved mean LLL and observed LLL of 0.5 was necessary to cause a lack of significant difference between treatment groups (-0.10 (-0.22 to 0.03)) (Table S3, Figure S1B).

Including auxiliary variables potentially predictive of missingness in the outcome had little effect on the obtained differences in in-stent LLL and in-segment LLL described above (Table S3)

**Table S2.** Baseline characteristics of the NITRATE-OCT trial for patients included in outcome analysis and those excluded from analysis. P-values comparing characteristics between included and excluded patients come from Student’s unpaired t-tests (continuous variables), Chi-square tests (categorical variables with >10 in all cells), or Fisher’s exact tests (categorical variables with ≤10 in any cell). BP, blood pressure; BPM, beats per minute; CABG, coronary artery bypass graft; CAD, coronary artery disease; CCS, Canadian Cardiology Society angina classification; COPD, chronic obstructive airways disease; CVA, cerebrovascular accident; MI, myocardial infarction; PCI, percutaneous coronary intervention; PVD, peripheral vascular disease; SD, standard deviation; TIA, transient ischaemic attack.

|  | **Included (*n*=208)** | **Excluded (*n*=92)** | **Comparison (p-value)** |
| --- | --- | --- | --- |
| Age (years) (mean ± SD) | 60.80 ± 9.19 | 63.13 ± 9.50 | ***0.046*** |
| Sex (Male) | 184 (88.5%) | 77 (83.7%) | 0.26 |
| Ethnicity |  |  | 0.88 |
| Caucasian | 162 (77.9%) | 75 (81.5%) |  |
| African-Caribbean | 15 (7.2%) | 5 (5.4%) |  |
| East Asian | 3 (1.4%) | 2 (2.2%) |  |
| South Asian | 28 (13.5%) | 10. (10.9%) |  |
| Indian | 15 (7.2%) | 5 (5.4%) |  |
| Bangladeshi | 3 (1.4%) | 0 |  |
| Pakistani | 10 (4.8%) | 5 (5.4%) |  |
| Diabetes mellitus | 43 (20.7%) | 23 (25.0%) | 0.40 |
| Type I | 2 (1.0%) | 0 |  |
| Type II | 41 (19.7%) | 23 (25.0%) |  |
| Body-mass index (kg/m^2^) (mean ± SD) | 28.96 ± 4.32 | 28.68 ± 5.21 | 0.64 |
| *Missing* | *2* | *3* |  |
| Hypertension | 150 (72.1%) | 71 (77.2%) | 0.36 |
| Hypercholesterolaemia | 151 (72.6%) | 65 (70.7%) | 0.73 |
| Previous MI | 79 (38.0%) | 32 (34.8%) | 0.60 |
| Previous PCI | 70 (33.7%) | 32 (34.8%) | 0.85 |
| Previous CABG | 9 (4.3%) | 5 (5.4%) | 0.77 |
| Current Smoker | 39 (18.8%) | 16 (17.4%) | 0.78 |
| Previous Smoker | 93 (44.7%) | 46(50.0%) | 0.40 |
| PVD | 11 (5.3%) | 10 (10.9%) | *0.090* |
| CVA/TIA |  |  |  |
| NYHA |  |  | 0.35 |
| Class I | 9 (4.5%) | 7 (7.7%) |  |
| Class II | 8 (4.0%) | 6 (6.6%) |  |
| Class III | 1 (0.5%) | 1 (1.1%) |  |
| *Missing* | *7* | *1* |  |
| CCS |  |  | 0.56 |
| CCS I | 37 (17.9%) | 18 (19.8%) |  |
| CCS II | 71 (34.3%) | 34 (37.4%) |  |
| CCS III | 93 (44.9%) | 39 (42.9%) |  |
| CCS IV | 5 (2.4%) | 0 |  |
| *Missing* | *1* | *1* |  |
| Asthma | 24 (11.5%) | 6 (6.5%) | 0.21 |
| COPD | 11 (5.3%) | 7 (7.6%) | 0.44 |
| Previous History of CAD | 104 (50.0%) | 51 (55.4%) | 0.39 |
| Heart rate (BPM) (mean ± SD) | 67.72 ± 13.02 | 69.20 ± 13.47 | 0.38 |
| *Missing* | *6* | *6* |  |
| Systolic BP (mmHg) (mean ± SD) | 135.41 ± 15.89 | 140.39 ± 18.39 | ***0.017*** |
| *Missing* | *11* | *6* |  |
| Diastolic BP (mmHg) (mean ± SD) | 77.78 ± 9.94 | 79.15 ± 10.20 | 0.29 |
| *Missing* | *11* | *6* |  |
| Culprit Vessel |  |  | 0.49 |
| Left main stem | 0 | 0 |  |
| Left anterior descending | 92 (44.2%) | 29 (46.0%) |  |
| First diagonal | 5 (2.4%) | 0 |  |
| Intermediate Artery | 4 (1.9%) | 1 (1.6%) |  |
| Circumflex | 38 (18.3%) | 7 (11.1%) |  |
| Obtuse Marginal | 7 (3.4%) | 1 (1.6%) |  |
| Right coronary | 62 (29.8%) | 25 (39.7%) |  |
| *Missing* | *0* | *29* |  |

**Table S3:** Key results from the sensitivity analysis examining the effect of missingness on the difference in late lumen loss between Inorganic Nitrate and Placebo treatment groups. LLL, late lumen loss.

| Difference between unobserved and observed mean LLL | Difference between unobserved and observed mean LLL in which treatment groups | Difference in LLL between Inorganic Nitrate and Placebo groups (95% CI) from | |
| --- | --- | --- | --- |
|  |  | **Pattern mixture model** | **Pattern mixture model with auxiliary variables** |
| *In-stent LLL* | | | |
| -0.5 | Both treatment groups | -0.13 (-0.24 to -0.03) | -0.13 (-0.24 to -0.03) |
|  | Placebo group only | 0.01 (-0.09 to 0.11) | 0.01 (-0.09 to 0.11) |
|  | Inorganic nitrate group only | -0.30 (-0.40 to -0.20) | -0.30 (-0.40 to -0.20) |
| -0.2 | Both treatment groups | -0.15 (-0.24 to -0.05) | -0.15 (-0.24 to -0.05) |
|  | Placebo group only | -0.09 (-0.18 to 0.00) | -0.09 (-0.18 to 0.00) |
|  | Inorganic nitrate group only | -0.21 (-0.31 to -0.12) | -0.21 (-0.31 to -0.12) |
| 0 (baseline) | Both treatment groups | -0.16 (-0.25 to -0.06) | -0.15 (-0.25 to -0.06) |
|  | Placebo group only | -0.16 (-0.25 to -0.06) | -0.15 (-0.25 to -0.06) |
|  | Inorganic nitrate group only | -0.16 (-0.25 to -0.06) | -0.15 (-0.25 to -0.06) |
| 0.2 | Both treatment groups | -0.16 (-0.26 to -0.07) | -0.16 (-0.26 to -0.07) |
|  | Placebo group only | -0.22 (-0.31 to -0.13) | -0.22 (-0.31 to -0.13) |
|  | Inorganic nitrate group only | -0.10 (-0.19 to 0.00) | -0.10 (-0.19 to 0.00) |
| 0.5 | Both treatment groups | -0.18 (-0.28 to -0.07) | -0.17 (-0.28 to -0.07) |
|  | Placebo group only | -0.32 (-0.42 to -0.22) | -0.32 (-0.42 to -0.22) |
|  | Inorganic nitrate group only | -0.01 (-0.11 to 0.09) | -0.01 (-0.11 to 0.09) |
| *In-segment LLL* | | | |
| -0.5 | Both treatment groups | -0.22 (-0.35 to -0.09) | -0.22 (-0.35 to -0.09) |
|  | Placebo group only | -0.08 (-0.20 to 0.05) | -0.08 (-0.20 to 0.05) |
|  | Inorganic nitrate group only | -0.38 (-0.51 to -0.26) | -0.38 (-0.51 to -0.26) |
| -0.2 | Both treatment groups | -0.23 (-0.35 to -0.11) | -0.23 (-0.35 to -0.11) |
|  | Placebo group only | -0.18 (-0.29 to -0.06) | -0.18 (-0.29 to -0.06) |
|  | Inorganic nitrate group only | -0.30 (-0.42 to -0.18) | -0.30 (-0.42 to -0.18) |
| 0 (baseline) | Both treatment groups | -0.24 (-0.36 to -0.12) | -0.24 (-0.36 to -0.12) |
|  | Placebo group only | -0.24 (-0.36 to -0.12) | -0.24 (-0.36 to -0.12) |
|  | Inorganic nitrate group only | -0.24 (-0.36 to -0.12) | -0.24 (-0.36 to -0.12) |
| 0.2 | Both treatment groups | -0.25 (-0.37 to -0.13) | -0.25 (-0.37 to -0.13) |
|  | Placebo group only | -0.31 (-0.43 to -0.19) | -0.31 (-0.43 to -0.19) |
|  | Inorganic nitrate group only | -0.18 (-0.30 to -0.07) | -0.18 (-0.30 to -0.06) |
| 0.5 | Both treatment groups | -0.26 (-0.39 to -0.13) | -0.26 (-0.39 to -0.13) |
|  | Placebo group only | -0.40 (-0.53 to -0.28) | -0.40 (-0.53 to -0.28) |
|  | Inorganic nitrate group only | -0.10 (-0.22 to 0.03) | -0.10 (-0.22 to 0.03) |

**Figure S1:** Key results from the sensitivity analysis examining the effect of missingness on the difference in late lumen loss between Inorganic Nitrate and Placebo treatment groups. LLL, late lumen loss.

**
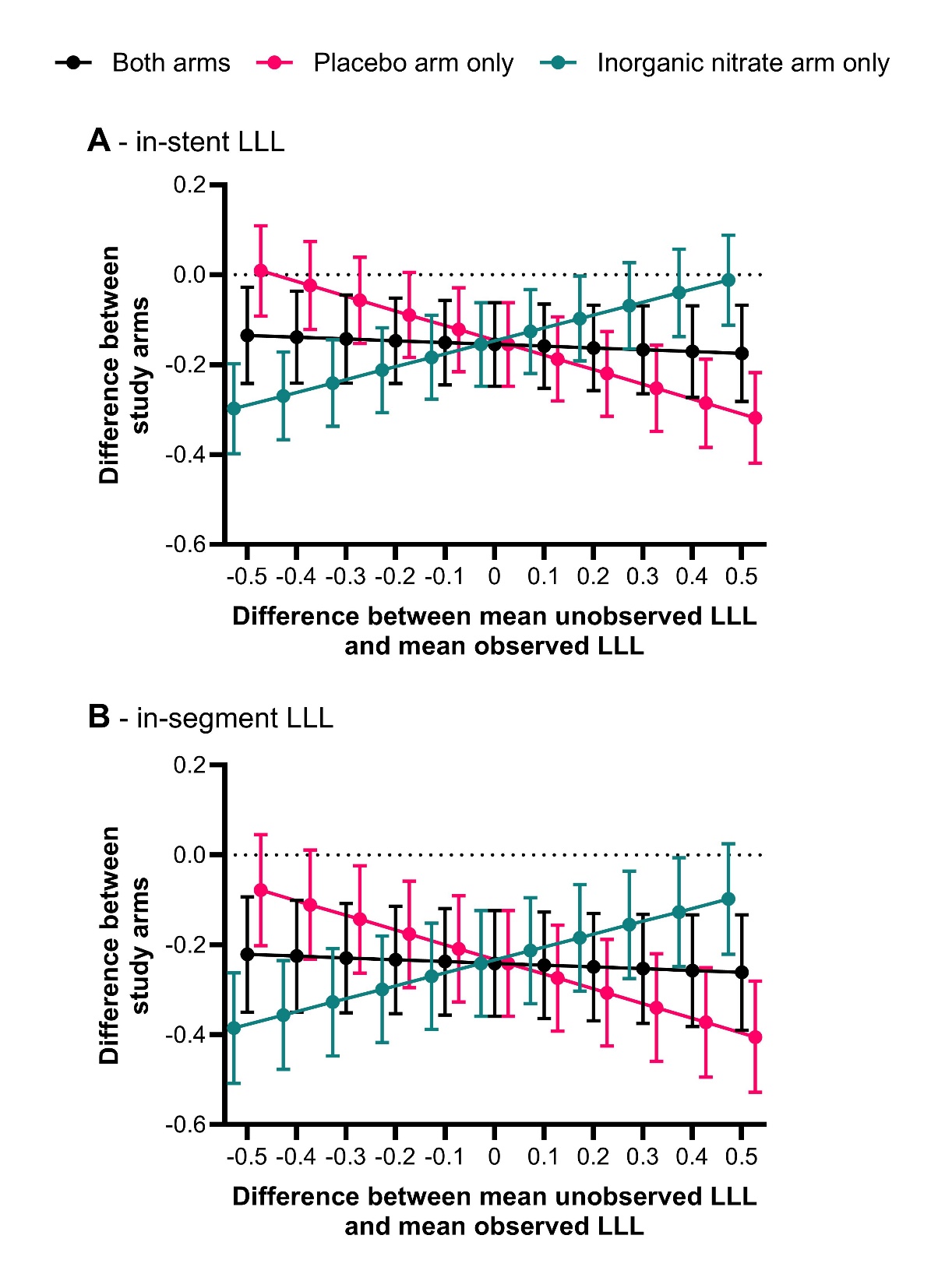
**

**Other analyses regarding the primary outcome**

**Statistical analysis methods**

The main linear regression analyses (Table 3) for in-stent LLL and in-segment LLL adjusted for diabetes and baseline MLD. Diabetes status was included as this was a stratification variable. Baseline MLD was included to control for the innate variability in vessel dimensions which could influence the extent of LLL.

A further regression analysis (called “fully adjusted”) adjusted for a full complement of important baseline covariates, in order to correct for any chance imbalances between randomisation groups, and to improve the precision of the treatment effect estimates. Covariates included in the fully adjusted model were: diabetes, baseline MLD, previous bypass surgery and procedural characteristics (culprit vessel, access site, DES use, number of stents, total stent length, and mean stent diameter). Procedural success was not included despite being described in the statistical analysis plan as all patients undergoing PCI experienced procedural success.

Subgroup analyses for primary endpoint variables within the ITT (Intention To Treat) population were pre-specified in the statistical analysis plan. Stratified linear regression models 1) adjusting for diabetes and baseline MLD and 2) fully adjusted were completed for subgroups based on baseline organic nitrate use (as part of routine therapy), i.e. no organic nitrate (n=87) vs organic nitrate use (n=121). Since all but one patient received a drug-eluting stent, we were not able to conduct the analysis based on stent type i.e. BMS vs DES.

Further per-protocol analyses of the primary and secondary endpoints were performed. These analyses were conducted on the dataset for LLL excluding all scans deemed unsuitable by the Independent CoreLab for QCA analysis.

**Table S4:** Intention to treat (ITT) analysis of the primary end-point using quantitative coronary angiography (QCA) of patients recruited into the NITRATE-OCT trial. This Table shows results of the linear regression models presented in Table 3 and further results from the fully-adjusted linear regression analysis model including all covariates: diabetes, baseline MLD, previous bypass surgery and procedural characteristics (culprit vessel, access site, DES use, number of stents, total stent length, and mean stent diameter. The estimated difference between treatment groups relates to the difference in Treatment Group vs Placebo Group (reference group). CI, confidence interval.

|  | Adjusted for diabetes and baseline MLD (results from Table 3) | | Fully-adjusted | |
| --- | --- | --- | --- | --- |
|  | Estimate difference (95% CI) | *P* value | Estimate difference (95% CI) | *P* value |
| In-stent late lumen loss (mm) | **-0.16 (-0.25, -0.06)** | **0.001** | **-0.16 (-0.25, -0.07)** | **<0.001** |
| In-segment late lumen loss (mm) | **-0.24 (-0.36, -0.12)** | **<0.001** | **-0.22 (-0.33, -0.11)** | **<0.001** |

**Figure S2.** Histogram showing the distribution of in-stent LLL in (A) the placebo-treated group and (B) in the inorganic nitrate-treated group


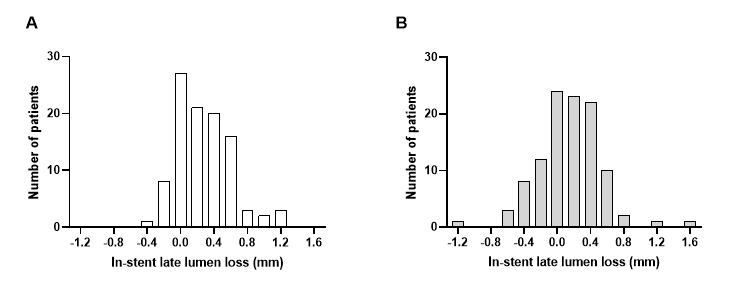


**Table S5:** Sub-group analysis of quantitative coronary angiography (QCA) of patients recruited into the NITRATE-OCT trial, stratified by baseline use of organic nitrate (No organic nitrate [n=87] vs organic nitrate [n=121]). Two different models are performed: analysis adjusted for diabetes and baseline MLD and fully adjusted analysis with adjustment for all covariates (as seen in Table S4). The estimated difference between treatment groups relates to the difference in Treatment Group vs Placebo Group (reference group). CI, confidence interval.

|  | Observed Mean ± SD | | Comparison between treatments | | | |
| --- | --- | --- | --- | --- | --- | --- |
|  |  |  | Adjusted for diabetes and baseline MLD | | Fully-adjusted | |
|  | Inorganic Nitrate | Placebo | Estimated difference (95% CI) | *P* value | Estimated difference (95% CI) | *P* value |
| **All patients** | *n*=107 | *n*=101 |  | | | |
| In-stent late lumen loss (mm) | 0.09 ± 0.38 | 0.24 ± 0.33 | **-0.16 (-0.25, -0.06)** | **0.001** | **-0.16 (-0.25, -0.07)** | **<0.001** |
| In-segment late lumen loss (mm) | 0.02 ± 0.52 | 0.26 ± 0.37 | **-0.24 (-0.36, -0.12)** | **<0.001** | **-0.22 (-0.33, -0.11)** | **<0.001** |
| **No organic Nitrate** | *n*=46 | *n*=41 |  | | | |
| Baseline in-stent MLD (mm) | 2.69 ± 0.52 | 2.72 ± 0.41 |  | | | |
| Baseline in-segment MLD (mm) | 2.37 ± 0.51 | 2.46 ± 0.47 |  |  |  |  |
| Follow up in-stent MLD (mm) | 2.53 ± 0.54 | 2.45 ± 0.41 |  |  |  |  |
| Follow up in-segment MLD (mm) | 2.37 ± 0.61 | 2.13 ± 0.56 |  |  |  |  |
| In-stent late lumen loss (mm) | 0.15 ± 0.34 | 0.27 ± 0.32 | -0.11 (-0.24, 0.03) | 0.113 | -0.14 (-0.29, 0.00) | 0.055 |
| In-segment late lumen loss (mm) | -0.00 ± 0.43 | 0.33 ± 0.44 | **-0.32 (-0.50, -0.13)** | **0.001** | **-0.30 (-0.50, -0.11)** | **0.003** |
| **Organic Nitrate** | *n*=61 | *n*=60 |  | | | |
| Baseline in-stent MLD (mm) | 2.69 ± 0.48 | 2.61 ± 0.45 |  | | | |
| Baseline in-segment MLD (mm) | 2.42 ± 0.62 | 2.34 ± 0.48 |  |  |  |  |
| Follow up in-stent MLD (mm) | 2.64 ± 0.56 | 2.39 ± 0.48 |  |  |  |  |
| Follow up in-segment MLD (mm) | 2.38 ± 0.64 | 2.12 ± 0.48 |  |  |  |  |
| In-stent late lumen loss (mm) | 0.05 ± 0.40 | 0.22 ± 0.33 | **-0.19 (-0.32, -0.06)** | **0.004** | **-0.20 (-0.32, -0.08)** | **0.001** |
| In-segment late lumen loss (mm) | 0.04 ± 0.58 | 0.22 ± 0.31 | **-0.20 (-0.36, -0.04)** | **0.012** | **-0.18 (-0.33, -0.03)** | **0.022** |

**Figure S3.** Modified Forest plot of the treatment effect in subgroups of patients recruited into the NITRATE-OCT trial. Groups are all patients (n=208), patients with no baseline use of organic nitrate (n=87) and patients with baseline of organic nitrate (n=121). Estimated differences come from models adjusted for diabetes and baseline MLD described in Table S5. Interaction p-values come from comparable models adjusting for diabetes and baseline MLD with an additional interaction between Treatment Group and baseline use of organic nitrate. Dotted line shows no effect point. Bold line shows the overall treatment effect point.


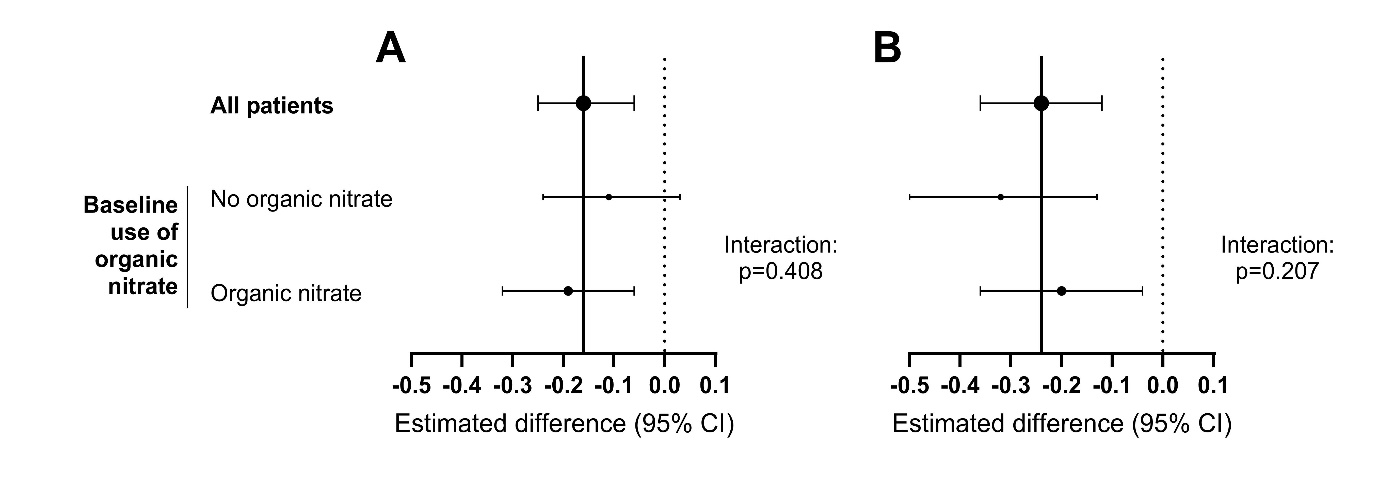


**Table S6.** **Quantitative coronary angiography (QCA).** Per protocol QCA analysis. This per protocol analysis includes only those scans deemed of sufficient quality by the Independent CoreLab. Data shown as mean ± SD or median (IQR) if non-normally distributed. P-value calculated by Student’s unpaired T test or Mann-Whitney test if non-normally distributed for baseline and follow-up parameters or linear regression models adjusted for diabetes and baseline MLD or fully adjusted analysis for all covariates (as seen in Table S4). The estimated difference between treatment groups relates to the difference in Treatment Group vs Placebo Group (reference group). CI, confidence interval. MLD-minimum lumen diameter.

|  | Observed Mean ± SD /Median (IQR) | | Comparison between treatments | | | |
| --- | --- | --- | --- | --- | --- | --- |
|  | Inorganic Nitrate (*n*=99) | Placebo (*n*=89) | Estimated difference (95% CI) | *P* value | Estimated difference (95% CI) | *P* value |
|  | | | **Unadjusted differences (t-tests/Mann Whitney U tests)** | | | |
| **Data from Table 4** | | | | | | |
| Baseline stent diameter (mm) | 3.21 ± 0.47 | 3.14 ± 0.41 | 0.07 (-0.06, 0.19) | 0.304 |  | |
| Baseline stent length (mm) | 24.8 (17.5-35.5) | 23.0 (18.2-33.6) | - | 0.704 |  |  |
| Baseline proximal reference stent diameter (mm) | 3.39 ± 0.65 | 3.34 ± 0.55 | 0.05 (-0.13, 0.22) | 0.601 |  |  |
| Baseline distal reference stent diameter (mm) | 2.69 ± 0.53 | 2.67 ± 0.48 | 0.02 (-0.13, 0.16) | 0.839 |  |  |
| Baseline in-stent diameter stenosis (%) | 12.1 (7.8-16.2) | 11.9 (8.0-16.9) | - | 0.593 |  |  |
| Baseline proximal maximum in-segment diameter (mm) | 3.79 ± 0.77 | 3.72 ± 0.76 | 0.07 (-0.15, 0.29) | 0.525 |  |  |
| Baseline distal maximum in-segment diameter (mm) | 2.97 ± 0.75 | 3.09 ± 0.70 | -0.12 (-0.33, 0.09) | 0.275 |  |  |
| Baseline in-segment diameter stenosis (%) | 18.1 (11.4-23.9) | 18.0 (12.0-23.1) | - | 0.727 |  |  |
| Follow up diameter stenosis in-stent (%) | 14.5 (10.0-19.3) | 14.4 (9.2-20.7) | - | 0.811 |  |  |
| Follow up diameter stenosis segment (%) | 17.6 (11.0-23.7) | 18.9 (11.3-25.3) | - | 0.476 |  |  |
| **Data from Table 3** | | | | | | |
| Baseline in-stent MLD (mm) | 2.72 ±0.49 | 2.67 ± 0.40 | 0.05 (-0.08, 0.18) | 0.229 |  | |
| Baseline in-segment MLD (mm) | 2.45 ± 0.56 | 0.41 ± 0.43 | 0.04 (-0.20, 0.19) | 0.586 |  |  |
| Follow up in-stent MLD (mm) | 2.60 ± 0.56 | 2.42 ± 0.43 | **0.18 (0.03, 0.32)** | **0.016** |  |  |
| Follow up in-segment MLD (mm) | 2.39 ± 0.62 | 2.14 ± 0.46 | **0.26 (0.10, 0.41)** | **0.002** |  |  |
|  | | | **Adjusted differences (linear regression models)** | | | |
|  |  |  | Adjusted for diabetes and baseline MLD | | Fully-adjusted | |
| In-stent late lumen loss (mm) | 0.12 ± 0.38 | 0.25 ± 0.33 | **-0.15 (-0.25, -0.05)** | **0.005** | **-0.16 (-0.25, -0.07)** | **0.001** |
| In-segment late lumen loss (mm) | 0.06 ±0.50 | 0.27 ± 0.37 | **-0.23 (-0.35, -0.11)** | **<0.001** | **-0.22 (-0.34, -0.11)** | **<0.001** |

**Analysis of Major adverse cardiac events (MACE)**

MACE is an exploratory endpoint in this study. Overall, at 2 years following recruitment into the study, 27 patients suffered MACE (7 deaths (2 in the inorganic nitrate group and 5 in the placebo group), 7 recurrent myocardial infarction (1 NSTEMI in the inorganic nitrate group and 6 (2 STEMIs and 4 NSTEMIs) in the placebo group) and 13 unscheduled revascularizations (6 (PCI) in the inorganic nitrate group and 7 (1 PCI and 6 CABG) in the placebo group)). 1 of the deaths in the inorganic nitrate group was due to trauma and the other cause of death unknown. In the placebo group, 1 patient died following mitral valve repair, 2 patients died following cancer (mesothelioma and leukaemia) and the causes of death of the other 2 patients unknown.

**Table S7** MACE data at 6, 12 and 24 months. Statistical significance is demonstrated using a Log-Rank test. MACE, major adverse cardiac events. ITT -intention to treat; PP-per protocol; MI-myocardial infarction; TVR- target vessel revascularisation

| **MACE** | **Inorganic Nitrate (ITT-150)** | **Placebo (ITT-150)** | **Log-Rank test** | **Inorganic Nitrate (PP-138)** | **Placebo**  **(PP-134)** | **Log-Rank test** |
| --- | --- | --- | --- | --- | --- | --- |
| **6 months** | 5 | 2 | 0.2399 | 5 | 2 | 0.2629 |
| Death | 0 | 1 |  | 0 | 1 |  |
| MI | 1 | 1 |  | 1 | 1 |  |
| TVR | 4 | 0 |  | 4 | 0 |  |
| ST | 0 | 0 |  | 0 | 0 |  |
| **12 months** | 7 | 8 | 0.8140 | 7 | 8 | 0.9511 |
| Death | 0 | 2 |  | 0 | 2 |  |
| MI | 1 | 1 |  | 1 | 1 |  |
| TVR | 6 | 5 |  | 6 | 5 |  |
| ST | 0 | 0 |  | 0 | 0 |  |
| **24 months** | 9 | 18 | 0.069 | 9 | 18 | 0.0491 |
| Death | 2 | 5 |  | 2 | 5 |  |
| MI | 1 | 6 |  | 1 | 6 |  |
| TVR | 6 | 7 |  | 6 | 7 |  |
| ST | 0 | 0 |  | 0 | 0 |  |

Note: No patients excluded from the per protocol (PP) analysis experienced MACE events.

**Analysis of the influence of inorganic nitrate on blood pressure**

**Statistical analysis methods**

Change in systolic, ad diastolic blood pressure and heart rate was calculated for each patient as parameter at follow-up – parameter at baseline. This change in parameter was compared between treatment groups using Student’s unpaired t-tests.

Examination of the normality of the change in each parameter (to meet the assumptions of the t-test) demonstrated that the change in heart rate in both treatment groups was negatively skewed (inorganic nitrate skew: -2.9; placebo skew: -1.1) invalidating the t-test assumptions. This was deemed to be due to extreme reductions in heart rate seen in 2 patients in the inorganic nitrate group (changes in heart rate of -48bpm and -87bpm) and 1 patient in the placebo group (change in heart rate of -37bpm). Using the rule that outliers can be considered values that fall outside of the range mean ± 3 standard deviations, all three of these patients could be excluded from the data along with 1 extra patient on inorganic nitrate with an extreme increase in heart rate (change in heart rate of 36bpm). Excluding these four patients changed the mean observed change in heart rate from -3.5 ± 12 in the inorganic nitrate group and -0.23 ± 7.6 in the placebo group to -2.7 ± 7.6 in the inorganic nitrate group and 0.12 ± 6.7 in the placebo group. Skewness was resolved by removing these four patients (inorganic nitrate skew: -0.13; placebo skew: -0.11).

**Figure S4** Inorganic nitrate lowers blood pressure. Figure shows impact of dietary nitrate upon systolic blood pressure (SBP), diastolic blood pressure (DBP) and heart rate. The change in SBP **(**A) DBP (B) and heart rate (C) at 6 months from baseline in the inorganic nitrate group compared with the placebo group is shown. Statistical analysis is shown for unpaired t-test comparison after exclusion of extreme outliers from the analysis of change in heart rate.


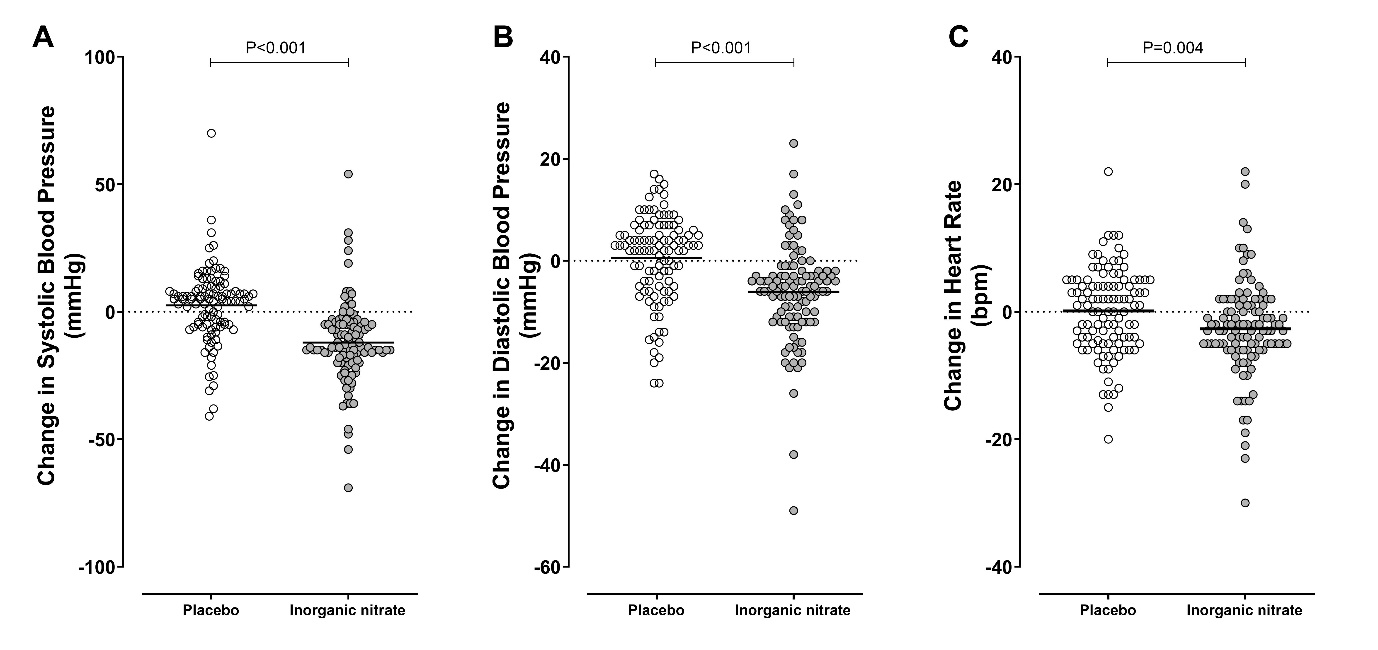


**Analysis of levels of nitrate and nitrite**

**Table S8. Measurement of nitrite and nitrate:** Plasma, urine, and saliva nitrate and nitrite levels measured in all samples available from recruited patients that completed the study, at baseline and after 6 months of treatment. The treatment was either nitrate-rich beetroot juice (intervention) 70 mL daily or nitrate-depleted juice (placebo) 70 mL daily, in patients with stable angina. Data expressed as mean ± SEM. *n*=92-96 (intervention) and *n*=86-102 (placebo). Statistical analysis was performed using Student’s paired T-test.

|  | | | | | | |
| --- | --- | --- | --- | --- | --- | --- |
|  | Inorganic nitrate  (*n*=92-96) | | *P* value | Placebo  (*n*=86-102) | | *P* value |
| **Biological sample** | **Baseline** | **6 months** |  | **Baseline** | **6 months** |  |
| Plasma nitrate (μmol/L) | 31.84 ± 2.59 | 195.71 ± 35.46 | <0.0001 | 34.09 ± 2.40 | 46.37 ± 5.01 | 0.0151 |
| Plasma nitrite (μmol/L) | 0.89 ± 0.11 | 1.80 ± 0.22 | 0.0001 | 1.15 ± 0.11 | 0.93 ± 0.11 | 0.0890 |
| Urine nitrate (μmol/L) | 898.37 ± 75.64 | 4465.40 ± 580.09 | <0.0001 | 965.13 ± 76.64 | 1142.64 ± 110.59 | 0.1478 |
| Urine nitrite (μmol/L) | 0.25 ± 0.03 | 0.81 ± 0.14 | 0.0001 | 0.37 ± 0.10 | 0.36 ± 0.05 | 0.8688 |
| Saliva nitrate (μmol/L) | 562.95 ± 71.84 | 3150.01 ± 343.73 | <0.0001 | 926.00 ± 186.53 | 620.12 ± 87.46 | 0.0332 |
| Saliva nitrite (μmol/L) | 374.90 ± 60.18 | 1327.45 ± 161.40 | <0.0001 | 484.73 ± 102.77 | 351.22 ± 56.30 | 0.0522 |

**Figure S5.** Inorganic nitrate treatment causes a small and non-symptomatic rise of methemoglobinemia levels. The figure shows the change in methemoglobinemia levels at 6 months relative to baseline in the dietary nitrate-treated (intervention group) compared to the placebo-treated group. Statistical analysis is shown for unpaired t-test comparison.

**Figure S6.** Correlation between dose of inorganic nitrate (mol/kg) received by patients taking either placebo or dietary inorganic nitrate compared to in-stent late lumen loss (LLL). The statistical significance (P) is demonstrated used linear regression analysis

**Table S9.** 6 month medical therapy. Values shown as number (%) unless otherwise stated. Abbreviations: ACEi: Angiotensin-converting enzyme; ARB: Angiotensin receptor blocker. DOAC: direct oral anticoagulants, GTN; Glyceryl tri-nitrate.

|  | Intervention (120)  (n=150) | Placebo (114)  (n=150) |  |
| --- | --- | --- | --- |
| **ACE inhibitor** | 73 (60.8%) | 53 (46.5%) | 0.060 |
| Ramipril | 54 (45.0%) | 47 (41.2%) | 0.789 |
| Enalapril | 3 (2.5%) | 0 (0.0%) | 0.248 |
| Perindopril | 4 (3.3%) | 2 (1.8%) | 0.685 |
| Lisinopril | 11 (9.2%) | 4 (3.5%) | 0.111 |
| Captopril | 1 (0.8%) | 0 (0.0%) | 1.000 |
|  |  |  |  |
| **ARB** | 17 (14.2%) | 26 (22.8%) | 0.091 |
| Lorsartan | 9 (7.5%) | 15 (13.2%) | 0.194 |
| Irbesartan | 2 (1.3%) | 1 (0.9%) | 1.000 |
| Candesartan | 6 (5.0%) | 9 (7.9%) | 0.427 |
| Valsartan | 0 (0.0%) | 1 (0.9%) | 0.484 |
|  |  |  |  |
| **Diuretic** | 14 (11.7%) | 8 (7.0%) | 0.265 |
| Furosemide | 3 (2.5%) | 5 (4.4%) | 0.492 |
| Bendroflumethiazide | 0 (0.0%) | 1 (0.9%) | 0.489 |
| Spironolactone | 3 (2.5%) | 0 (0.0%) | 0.247 |
| Eplerenone | 3 (2.5%) | 1 (0.9%) | 0.622 |
| Indapamide | 4 (3.3%) | 2 (1.8%) | 0.684 |
| Hydrochlorothiazide | 1 (0.8%) | 0 (0.0%) | 1.000 |
|  |  |  |  |
| **Calcium Channel Blocker** |  |  |  |
| Amlodipine | 30 (20.0%) | 28 (24.6%) | 1.000 |
|  |  |  |  |
| **b Blocker** | 87 (58.0%) | 87 (76.3%) | 0.339 |
| Bisoprolol | 82 (68.3%) | 80 (70.8%) | 0.554 |
| Atenolol | 2 (1.3%) | 4 (3.5%) | 0.431 |
| Metoprolol | 1 (0.8%) | 2 (1.8%) | 0.609 |
| Nebivilol | 1 (0.8%) | 0 (0.0%) | 1.000 |
| Propanolol | 0 (0.0%) | 1 (0.9%) | 0.480 |
| Other | 1 (0.8%) | 0 (0.0%) | 1.000 |
|  |  |  |  |
| **Statins** | 115 (95.8%) | 104 (91.2%) | 0.431 |
| Atorvastatin | 99 (82.5%) | 95 (83.3%) | 0.288 |
| Pravastatin | 2 (1.3%) | 0 (0.0%) | 0.499 |
| Rosuvastatin | 14 (11.7%) | 8 (7.0%) | 0.369 |
| Simvastatin | 0 (0.0%) | 1 (0.9%) | 0.475 |
